# Enhancing the Membranolytic Activity of *Chenopodium* *quinoa* Saponins by Fast Microwave Hydrolysis

**DOI:** 10.3390/molecules25071731

**Published:** 2020-04-09

**Authors:** Emmanuel Colson, Philippe Savarino, Emily J.S. Claereboudt, Gustavo Cabrera-Barjas, Magali Deleu, Laurence Lins, Igor Eeckhaut, Patrick Flammang, Pascal Gerbaux

**Affiliations:** 1 Organic Synthesis and Mass Spectrometry Laboratory (S²MOs), University of Mons - UMONS, 23 Place du Parc, 7000 Mons, Belgium; emmanuel.colson@umons.ac.be (E.C.); Philippe.savarino@umons.ac.be (P.S.); 2 Biology of Marine Organisms and Biomimetics Unit (BOMB), University of Mons - UMONS, 23 Place du Parc, 7000 Mons, Belgium; Emily.CLAEREBOUDT@umons.ac.be (E.J.S.C.); igor.eeckhaut@umons.ac.be (I.E.); Patrick.Flammang@umons.ac.be (P.F.); 3 Laboratory of Molecular Biophysics at Interfaces, TERRA Research Center, Gembloux Agro-Bio Tech, University of Liege, 5030 Gembloux, Belgium; magali.deleu@uliege.be (M.D.); l.lins@ulg.ac.be (L.L.); 4 Unidad de Desarrollo Tecnológico (UDT), Universidad de Concepción, Av. Cordillera 2634, Parque Industrial Coronel, Coronel P.O. Box 4051 mail 3, Región del BíoBío, Chile; g.cabrera@udt.cl

**Keywords:** saponin, quinoa, mass spectrometry, structure activity relationship, microwave heating

## Abstract

Saponins are plant secondary metabolites. There are associated with defensive roles due to their cytotoxicity and are active against microorganisms. Saponins are frequently targeted to develop efficient drugs. Plant biomass containing saponins deserves sustained interest to develop high-added value applications. A key issue when considering the use of saponins for human healthcare is their toxicity that must be modulated before envisaging any biomedical application. This can only go through understanding the saponin-membrane interactions. Quinoa is abundantly consumed worldwide, but the quinoa husk is discarded due to its astringent taste associated with its saponin content. Here, we focus on the saponins of the quinoa husk extract (QE). We qualitatively and quantitively characterized the QE saponins using mass spectrometry. They are bidesmosidic molecules, with two oligosaccharidic chains appended on the aglycone with two different linkages; a glycosidic bond and an ester function. The latter can be hydrolyzed to prepare monodesmosidic molecules. The microwave-assisted hydrolysis reaction was optimized to produce monodesmosidic saponins. The membranolytic activity of the saponins was assayed based on their hemolytic activity that was shown to be drastically increased upon hydrolysis. In silico investigations confirmed that the monodesmosidic saponins interact preferentially with a model phospholipid bilayer, explaining the measured increased hemolytic activity.

## 1. Introduction

For years, natural molecules have been a hot research topic, not only because of their diversity but also because of their incredible structural complexity that is associated with specific biological activities. Numerous pharmaceutical properties have been discovered for these compounds. In addition, the industrial interest in producing new green drugs has greatly increased. Amongst the relevant biomolecules, secondary metabolites, such as flavonoids, alkaloids and saponins, represent key target molecules for scientific research [1,2,3]. In particular, saponins are abundantly investigated for their surface-active properties, such as their amphiphilicity and their cytotoxicity [4,5]. Saponins are abundant secondary metabolites in plants [3,4,6] and were more recently detected in different marine invertebrates such as sea stars, holothurians and sponges [7,8,9,10]. Even if the saponin family of molecules is characterized by a huge chemical diversity [7,9,11], the saponin structure invariably associates two distinct moieties, an apolar steroidic or triterpenoidic aglycone and a polar oligosaccharidic part, named the glycone.

Based on their amphiphilic structure, saponins are active against numerous organisms such as bacteria, fungi or cancer cells [3,5,7,12]. Their mode of action is ascribed to their membranolytic activity [5,13,14]. It is indeed suggested that saponins interact with membrane sterols forming in-membrane saponin/sterol complexes. Upon extensive saponin molecule incorporation into the membrane, these complexes aggregate inducing the formation of pores. The concomitant membrane permeabilization ultimately leads to cell death [5,13,14,15]. In the last decade, the interaction between saponins and biological membranes was shown to strongly depend on the structure of the saponins [5,13,14,15,16]. Recently, using theoretical approaches, several groups have investigated the structure-activity relationship between saponins and biological membranes at the molecular level [5,13,17,18].

Structural characterization of saponin extracts is a challenging task because of the presence of numerous saponin molecules, with only subtle structural differences, in a given saponin extract making their characterization difficult [19]. Today, saponin characterization takes more and more advantage of the capabilities of mass spectrometry-based methods and LC-MS experiments are very efficient for saponin mixture characterization [6,9], often using accurate mass measurements (high-resolution MS–HRMS) and tandem mass spectrometry experiments (collision-induced dissociation–CID) [6,9,20]. We have reported the use of ion mobility spectrometry (IMS) [21,22] for saponin characterization [23,24]. We shown that isomeric saponins presenting different connectivities between the glycone and the aglycone moieties can be distinguished using IMS [24]. More recently, taking advantage of the high ion mobility resolution achieved on the newly-introduced cyclic ion mobility experimental setup [25], we succeeded in distinguishing regioisomeric and steroisomeric saponins from *Aesculus hippocastanum* [20,25].

**Figure 1:**
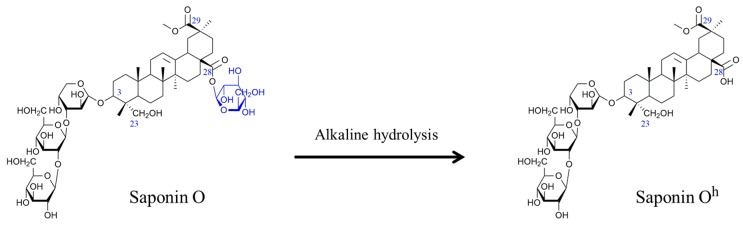
Alkaline hydrolysis of Saponin O to Saponin O^h^.

The present study focuses on the saponins contained in the husk of Chilean quinoa (*Chenopodium quinoa)*. Whereas quinoa is one of the most consumed food across the world due to its high nutritive value and the resistance of the crop to drought, cold and hard climates [6,26], the quinoa husk is discarded, mostly due to its astringent taste associated with its high saponin content [26]. Today, the quinoa husk is an abundant food industry waste that demands valorization to take advantage of the properties of its saponins. The quinoa saponins have already been described [6,26,27] with up to 70 compounds being detected by Madl et al. by combining GC-MS and LC-MS/MS [6]. These saponins are triterpenoid-based and the major aglycones found are phytolaccagenic acid, hederagin and oleanic acid that are often linked to two different oligosaccharides, attached on C_3_ and C_28_, leading to bidesmosidic molecules, see Figure 1 [6,26,27]. Whereas the oligosaccharidic chains appended on C_3_ are characterized by a certain diversity, a single glucose residue (Glc) is invariably present on C_28_ [6,27]. The biological activities of the quinoa saponin extracts have been studied and they are active against several types of bacteria, viruses and animals [26,27,28]. They are also used as an adjuvant in vaccines [29]. Recently, quinoa husk saponin extracts were subjected to an alkali treatment with 1M NaOH at 95–100 °C for 2 h to generate modified saponins with enhanced antibacterial [26], antifungal [30] and molluscicide [28] activities. Even if it is likely that alkali hydrolysis of quinoa saponins will mostly affect the C_28_ ester function, as proposed in Figure 1, no in-depth structural analysis was achieved in these contributions rendering the nature of the saponin molecules in the reaction mixtures only tentative. However, such an approach represents an elegant way to prepare modified saponin mixtures with enhanced biological activities [26].

In the current study, we report on the fast and quantitative bidesmosidic-to-monodesmosidic saponin conversion (Figure 1) using a microwave-assisted hydrolysis and the comparison between the cytotoxicity of the modified and the original saponins. The objectives of the present investigation are numerous and we intend to contribute: (i) to the establishment of the structure/activity relationship (SAR) of the saponins by specifically modifying their chemical structures and (ii) to the exploration of avenues for using quinoa husk by quantitatively and qualitatively establishing its saponin content. The determination of the cytotoxicity of the quinoa husk saponins, including chemical methods to modify their activity, will also be mandatory for future works exploring high added value applications. To achieve our objectives, we will rely on state-of-the-art mass spectrometry characterization, including LC-MS, CID and ion mobility, to fully identify the saponin molecules in the original and hydrolyzed extracts. As for a model cytotoxicity assay, we will determine the hemolytic activity of saponins, i.e., their propensity to lyse red blood cell membranes [5]. In addition, in silico investigations will be achieved using the IMPALA method to describe at a molecular level the insertion mechanism of saponins within an implicit membrane.

## 2. Results and Discussion

### 2.1. Saponin Identification and Quantification in the Quinoa Extract (QE)

**Figure 2:**
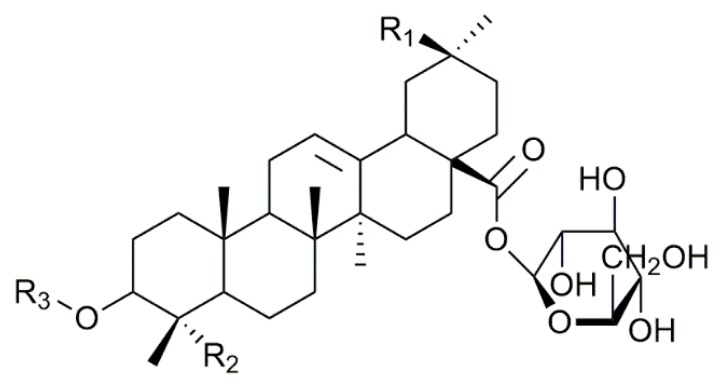
Schematic structure of the saponin molecules extracted from the quinoa husk. All the functions in R_1_, R_2_ and R_3_ are described in Table 1.

**Figure 3:**
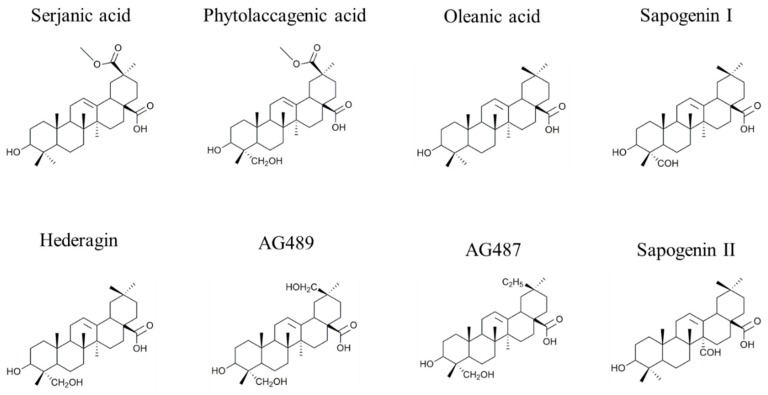
General structures of the sapogenins detected in *Chenopodium quinoa* extract in previous reports [6,28].

The saponin extract from the quinoa husk is analyzed using the MS-based protocol that was described previously [23,24] that combines MALDI-MS, HRMS, LC-MS, LC-MSMS and LC-IMS. Saponin identification is then achieved by comparing our MS data to the quinoa saponins qualitatively described by Madl et al. [6] and Kuljanabhagavad et al. [27]. *Chenopodium quinoa* husk saponins are bidesmosidic saponins systematically presenting a glucose residue at the C_28_ position (see Figure 1 and Figure 2), whereas, in the C_3_ oligosaccharide chain, two or three sugars, namely glucose (Glc), galactose (Gal), xylose (Xyl), Arabinose (Ara) and glucuronic acid (GlcA), are often present [6,27]. From the literature [6], different aglycone moieties are to be considered, i.e., oleanic acid, hederagin, AG489 (MW 488 u, C_30_H_48_O_5_), AG487 (MW 486 u, C_31_H_50_O_4_), serjanic acid and phytolaccagenic acid (see Figure 1). In addition, Kuljanabhagavad et al. also identified aglycones bearing a C(=O)H group at either C23 or C27 [27] (see Figure 3).

**Figure 4:**
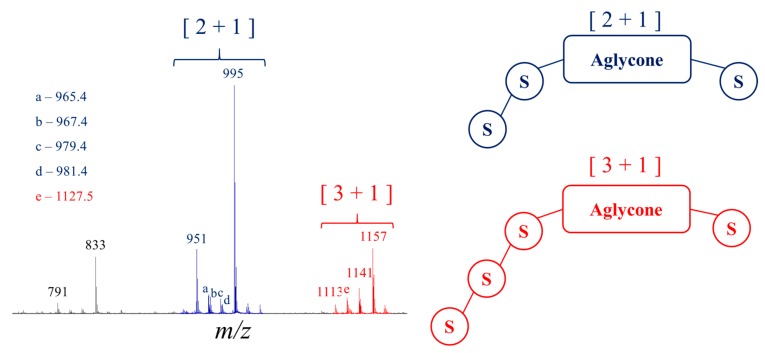
Mass spectrometry analysis of the quinoa saponin extract: MALDI-MS (+) spectrum (DHB/DMA as the matrix) and schematic representation of the [2 + 1] and [3 + 1] saponin structures. Ions detected at *m*/*z* 791 and 833 are fragment ions from the intact saponin ions. This is demonstrated using LC-MS analysis as shown in the SI.

Figure 4 presents the MALDI mass spectrum of the quinoa saponin extract. All the saponin molecules appear as sodium adducts, [M + Na]^+^, and basically two groups of MS signals are detected. The elemental compositions of the saponin ions are first confirmed based on accurate mass measurement, as presented in Table 1. Based on the measured mass-to-charge ratios (*m*/*z*), we detect 10 different elemental compositions that correspond to saponin ions (*vide infra*). The first group of saponin ions in the *m*/*z* 1100–1160 range includes four-saccharide saponins, whereas the saponins detected between *m*/*z* 950–1000 are three-saccharide saponins. The MALDI data must be complemented by LC-MS analysis to: (i) evaluate the presence of isomeric saponins, (ii) evaluate the monosaccharide sequence using LC-MSMS [23] and (iii) confirm the bidesmosidic nature of the detected saponins using LC-IMS [24]. In Table 1, we summarize all the MS data including the retention times in LC-MS and the saponin ion collision cross sections (^TW^Ω_N2->He_) as determined using ion mobility experiments [31]. By associating the HRMS data to the LC-MS and MSMS information, we detect and (partially) identify 12 saponins (see Table 1) including three original molecules (*shaded cells*). The saponin names come from the literature [6].

All the CID spectra (collision-induced dissociations) that helped us to identify the saponins are gathered in the supplementary information. In particular, the aglycone identifications in Figure 3 are based on the mass-to-charge ratios of the aglycone ions detected in the CID spectra in Figures S1–S24. Doing so, saponins containing oleanic acid, hederagin, AG489, serjanic acid and phytolaccagenic acid are detected. For the saponins observed at *m*/*z* 967, we here detect two different isomers that are barely distinguished using MS methods with similar CID spectra and identical collision cross sections, ^TW^CCS_N2⭢He_. They are only distinguishable upon liquid chromatography with a retention time (t_R_) difference close to 1 min, see Table 1. For the time being, no structural information is available to establish the corresponding structures, and isolation of the compounds to allow nuclear magnetic resonance measurements is out of the scope of the present study. As far as the *m*/*z* 965 and 1127 saponins are concerned, both saponin compositions are original and we propose that they are only distinguished by the presence of an additional monosaccharide on the R_3_ sugar chain in the latter. This is clearly observed using LC-MSMS experiments (see SI1a-x). In these CID spectra, the sapogenin ions are detected at *m*/*z* 487. The sapogenin ions also suffer, upon extensive ion activation, consecutive losses of two water molecules and carbon monoxide (28 u). In their publication, Madl et al. also detected a 28 u loss they assigned to C_2_H_4_ loss [6] allowing them to introduce AG487 (Figure 1) as a new sapogenin [6]. Based on our HRMS measurements, we rather propose that the corresponding sapogenin must present a C(=O)H group at C23 or C27 like the saponins detected by Kuljanabhagavad et al. and characterized using NMR [28]. Again, compared to the proposed sapogenins I and II in [27], our HRMS measurements points to the presence of an additional oxygen atom on the aglycone leading to the detection of a new sapogenin, that should deserve further characterization before being unambiguously identified (*question mark* in Table 1). Isolation of these new aldehyde-containing saponins will represent a time-consuming task and we are now rather exploring the possibility to conduct liquid chromatography-Infrared Ion Spectroscopy to highlight the presence of the aldehyde group since infrared spectroscopy is known to be sensitive to the presence of C=O bonds [32]. 

**Figure 5:**
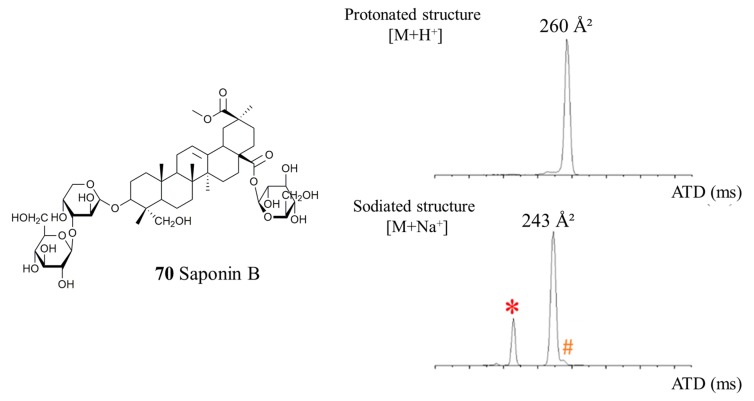
Ion mobility experiments on quinoa saponin ions: Arrival Time Distributions (ATD) of protonated (*m*/*z* 973) and sodiated (*m*/*z* 995) Saponin B. The ^TW^CCS_N2⭢He_ presented at the top of the signals are calculated using the calibration procedure presented in the experimental section. For the instrumental conditions used in the ion mobility experiments, see the Experimental Section. The signals marked with (*,^#^) correspond to the doubly-charged [M + 2Na]^2+^ ions and to a fragment of ionized saponin O–The loss of glucose at C28 upon ion activation from [Saponin O + Na]^+^ generates [Saponin O^h^ + Na]^+^ (see Figure 1) that is the monodesmosidic isomer of [Saponin B + Na]^+^.

Based on LC-MSMS experiments, the distinction between the monodesmosidic and the bidesmosidic topologies is difficult to achieve [6]. In a recent report [24], we observed that [M + H]^+^ and [M + Na]^+^ saponin ions present different mobilities depending on whether the molecule is monodesmosidic or bidesmosidic. Indeed, the [M + Na]^+^ bidesmosidic ions are always significantly more compact than their [M + H]^+^ homologues, whereas for the monodesmosidic molecules the CCS are almost identical for the [M + H]^+^ and [M + Na]^+^ ions [24]. As a typical example (see Figure 5), the saponin B ions, detected at *m*/*z* 973 and 995 for [M + H]^+^ and [M + Na]^+^ ions, respectively, present significantly different collision cross-sections, ^TW^CCS_N2⭢He_, at 260 and 243 Å^2^ respectively. In our previous work, we noted that a CCS difference, say ΔCCS = (CCS_[M + Na]_ − CCS_[M + H]_)/CCS_[M + H]_, of around 10% identifies a bidesmosidic structure [24]. As shown in Table 1, all the saponin molecules detected in the quinoa extract are confirmed to be bidesmosidic saponins using ion mobility experiments. Indeed, the averaged ΔCCS amounts to 7.4 ±1.3 Å^2^. Note that in the present paper, we will use the [x + y] symbolism to count the number of monosaccharide residues appended at C3 (x) and C28 (y). For instance, [2 + 1] and [3 + 0] respectively characterize trisaccharide saponins presenting bidesmosidic and monodesmosidic topologies.

**Figure 6:**
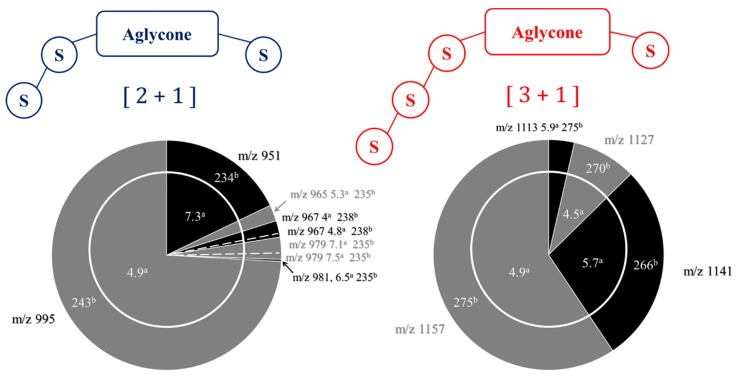
Molecular diversity of the three and four-sugar saponins from the quinoa extract. Integration of the MALDI-ToF, LC-MS(MS), and LC-IMS-MS data with (**a**) the retention times in LC (min) and (**b**) the collision cross sections in IMS (Å^2^). Section areas are associated with LC-MS semi-quantitative analysis using Hederacoside C as the internal standard and correspond to molar proportions (% in the quinoa husk extract). The relative proportions are also given in Table 1.

Using hederacoside C (Sigma-Aldrich) as an internal standard, we determine the mass proportions of all the saponins detected in the quinoa extract using LC-MS experiments, as presented in Table 1. Hederacoside C is commercially available and extracted from ivy leaves [34]. We also represent the semi-quantitative analysis results using sector diagrams that are constructed from mass spectrometry data [23], including LC retention times (t_R_), *m*/*z* ratios and ^TW^CCS_N2⭢He_ values, see Figure 6. This schematic representation has been recently introduced [23] to facilitate data interpretation since it allows for a direct and fast comparison, both in terms of compositions and relative proportions of the saponins in different extracts. Using the semi-quantitative analysis, we also estimate that the saponin content amounts to around 90% by weight of the quinoa extract.

### 2.2. Selectivity of the Microwave-Assisted Hydrolysis of Saponins

**Figure 7:**
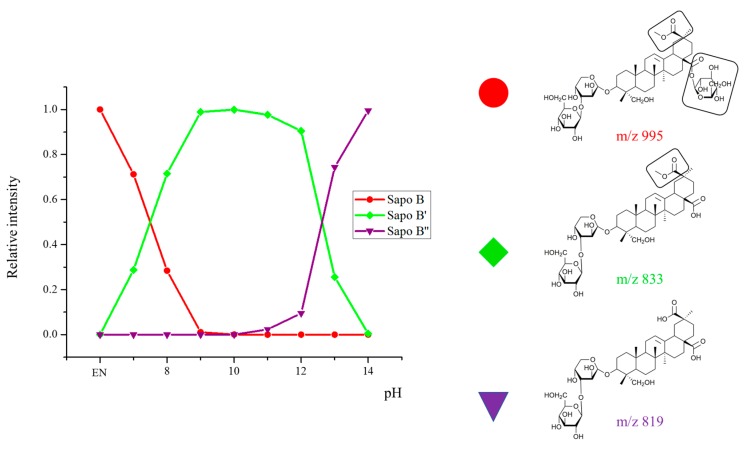
Microwave-assisted hydrolysis of the quinoa bidesmosidic saponins (5 min at 150 °C): influence of the pH on the extent of the consecutive hydrolysis reactions from Saponin B as determined using LC-MS. The given *m*/*z* ratios correspond to the [M + Na]^+^ ions, but for the estimation of the relative proportions, both the [M + H]^+^ and the [M + Na]^+^ ions have been considered.

As shown in Figure 1 and Figure 2, the bidesmosidic saponins of the quinoa extract present two different aglycone-monosaccharide linkages, i.e., a ketal-type glycosidic bond at C3 and an ester bond at C28. One of the objectives of the present study is to convert bidesmosidic saponins to their monodesmosidic form by withdrawing one of the oligosaccharide chains under microwave activation. The presence of the hydrolysable ester function is obviously targeted, and we envisage to use a basic hydrolysis to avoid hydrolysis of the glycosidic bonds [26]. However, other water-sensitive functions, such as the methyl ester functions at C_29_, in serjanic acid (Figure 3) for Saponins H, G and in phytolaccagenic acid (Figure 3) for Saponins B and O, are also present on the saponin molecules listed in Table 1. Using microwave heating, the tunable parameters are the pH, the temperature and the irradiation time. We decided to use 5 min irradiation time as the starting point for the microwave-assisted hydrolysis. For optimizing the hydrolysis conditions, we pay attention to the hydrolytic decompositions of Saponin B that is the most abundant saponin in the quinoa extract, as reported in Table 1. This saponin contains phytolaccagenic acid acid as the hydrolysable aglycone. Therefore, upon basic hydrolysis, we can expect two different hydrolysis products; i.e., saponin B’ (hydrolysis at C_28_–glucose loss) and saponin B” (hydrolysis at C_28_ and at C_29_–glucose and methanol losses) as presented in Figure 7. The targeted hydrolysis product is Saponin B’ (*m*/*z 833*) and is already known in the literature as esculentoside C, a compound that can be extracted from the roots of *Radix phytolaccae* [35]. To analyze the influence of the pH on the specificity of the hydrolysis reaction, we estimate the relative proportions of the Saponins B, B’ and B” using LC-MS experiments by considering the ion signal intensities of the [M + H]^+^ and [M + Na]^+^ for the three compounds. From Figure 7 starting from the saponin composition in the natural extract, we observe that the optimal pH is around 10.

**Figure 8:**
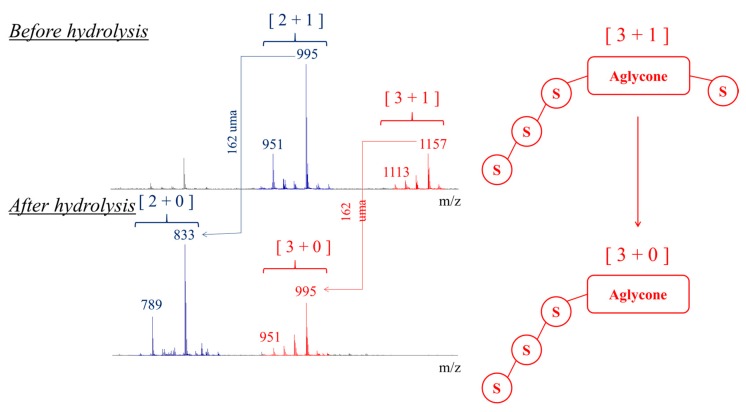
Microwave-assisted alkaline hydrolysis (5min, pH 10, 150 °C) of Chenopodium quinoa saponin extract: MALDI (+) mass spectra of (**a**) the saponin extract and (**b**) the hydrolyzed saponins.

**Figure 9:**
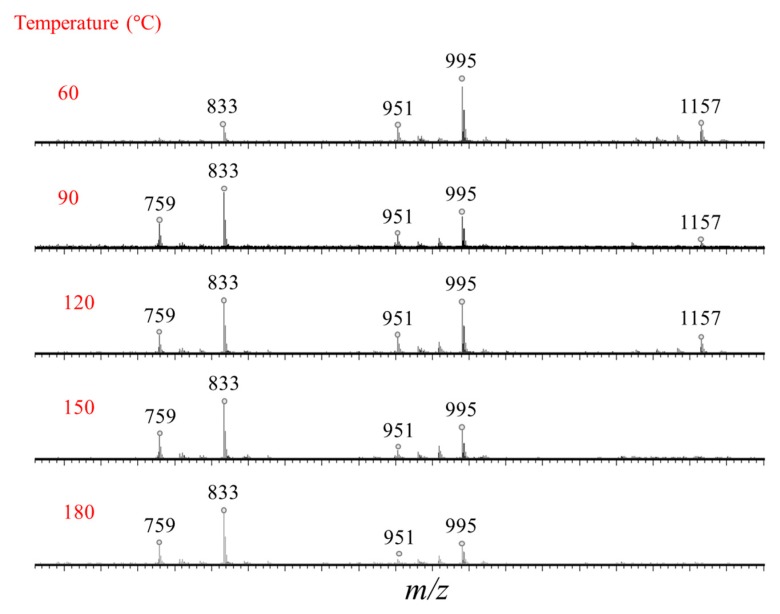
Microwave-assisted hydrolysis of the quinoa bidesmosidic saponins (5 min at pH 10): influence of the temperature (60,90,120,150,180 °C) on hydrolysis reactions from quinoa husk saponin determined by MALDI-MS.

The hydrolyzed saponin solution is then analyzed by MALDI-MS and the recorded mass spectrum is compared to the mass spectrum of the initial saponin solution in Figure 8. The influence of the temperature on the hydrolysis reaction progress is also monitored using MALDI analysis as presented in Figure 9 and we note that quantitative hydrolysis is obtained for 5 min irradiation around 150 °C at pH 10.

**Figure 10:**
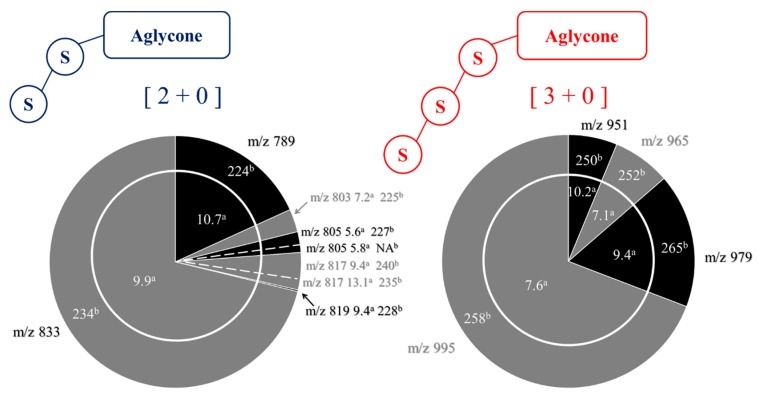
Microwave-assisted alkaline hydrolysis (5min, pH 10, 150 °C) of Chenopodium quinoa saponin extract: composition of the hydrolysate. Integration of the MALDI-ToF, LC-MS(MS), and LC-IMS-MS data with (**a**) the retention times in LC (min) and (**b**) the collision cross sections in IMS (Å^2^). Section areas are associated with LC-MS semi-quantitative analysis using Hederacoside C as the internal standard and correspond to molar proportions (% in the hydrolyzed quinoa husk extract). The relative proportions are also given in Table 2.

**Figure 11:**
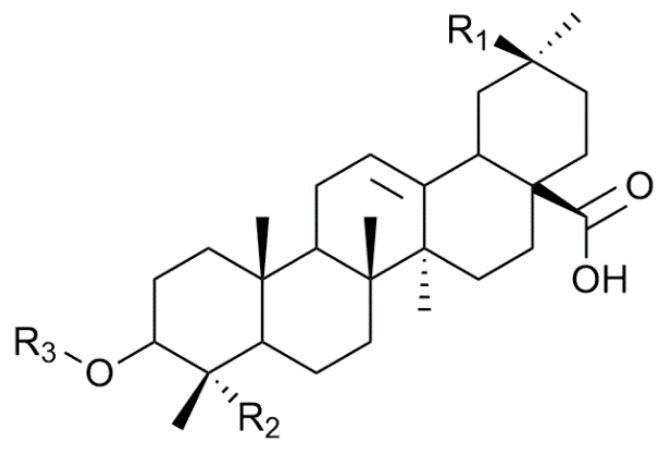
Schematic structure of the hydrolyzed saponins extracted from the quinoa husk. All the functions in R_1_, R_2_ and R_3_ are described in Table 2.

Figure 8b presents the MALDI mass spectrum of the hydrolysate obtained in those experimental conditions. We note the disappearance of the [3 + 1] saponin ions, whereas the signals at *m*/*z* 789 and 833 are now more intense. The C28 glucose loss upon hydrolysis converts [3 + 1] and [2 + 1] saponins into, respectively, [3 + 0] and [2 + 0] saponins, see Figure 8. The [2 + 0] saponin ions are detected at *m*/*z* 789 and 833, whereas the [3 + 0] ions and the [2 + 1] ions are isomers and detected in the *m*/*z* 950–995 range. Using LC-MS experiments, we can establish that the *m*/*z* 950–995 signals uniquely correspond to [3 + 0] saponins and that no residual [2 + 1] saponins are present. Indeed, as presented in the sector diagrams of Figure 10, the *m*/*z* 951, 965, 979 and 995 ions are each characterized by a single retention time when submitted to LC-MS separation. Ion mobility experiments further confirm the monodesmosidic nature of the produced saponins. As for a typical example, for the *m*/*z* 995 [M + Na]^+^ ions, their CCS appear significantly different, 243 Å^2^ (Figure 6) and 258 Å^2^ (Figure 10), for respectively the [2 + 1] and [3 + 0] topologies. In Table 2, the CCS for the [M + H]^+^ and [M + Na]^+^ ions are given allowing to calculate an average value for ΔCCS = −1.1 ± 2.0 Å^2^. This value can be compared to the ΔCCS for the non-hydrolyzed saponins in Table 1 (7.4 Å^2^) confirming the monodesmosidic nature of the saponins (see Figure 11) present in the hydrolysate. We will name the hydrolyzed saponins starting from the name of the parent molecules by just adding a superscript “h”. For instance, Saponin B becomes Saponin B^h^. All saponin structures are reported in Table 2.

Using Hederacoside C as an internal standard, we determine the mass proportions of all the saponins detected in the QE hydrolysate using LC-MS experiments. The results are presented in Table 2 and Figure 10. The direct comparison of the sector diagrams in Figure 6 and Figure 10 indicates that the hydrolysis reaction is quantitative and converts the natural saponins in their hydrolyzed monodesmosidic counterparts while conserving the relative proportions.

### 2.3. Activity Modulation–Hemolytic Activity Assay

**Figure 12:**
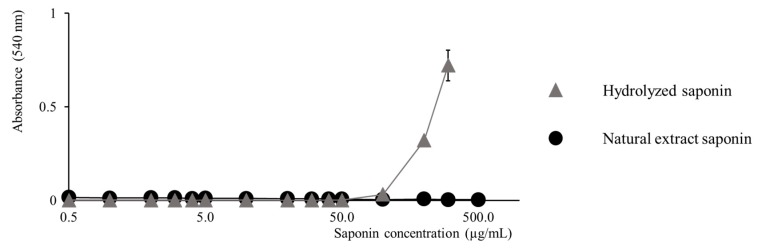
Estimation of the membranolytic property of the natural and hydrolyzed saponins via the hemolytic activity assay. Monitoring the free heme absorbance (540 nm) with regards to the increasing saponin concentration. Hydrolyzed saponins appear to be more cytotoxic than the non-hydrolyzed ones. Hemolytic activity experiments were performed in triplicates and the average data as well as the standard deviations are gathered in Table S1.

QE saponins have already been demonstrated to be active against bacteria, cancer cells and other organisms [26,28]. Evaluation of the cytotoxicity of saponins is often performed based on the hemolytic activity assay, i.e., determining the propensity of saponins to induce the rupture of the red blood cell membranes and the release of the free heme into the solution [5]. The hemolytic activity is then quantified using a colorimetric assay targeting the heme concentration in solution (absorbance at 540 nm) [5]. In Figure 12, we compare the evolution of the absorbance at 540 nm with regards to the increasing saponin extract concentration for the QE saponins and the hydrolyzed saponins. The concentration is expressed in µg of saponins per ml of the red blood cell solution suspension. We note that the QE saponins remain inactive even at a concentration of 500 µg/mL that corresponds to the solubility limit of the saponins in the red blood cell suspension. On the contrary, already at 100 µg/mL, the hydrolyzed saponins start to be active against red blood cells since the 540 nm absorbance steadily increases with increasing saponin concentration. The experiments with hydrolyzed saponins is limited to an upper concentration of 300 µg/mL for solubility reasons.

The observation of the increased membranolytic activity of the hydrolyzed saponins when compared to the natural molecules can be readily associated with the monodesmosidic topology of the hydrolyzed molecules allowing them to interact more strongly with the cell membrane. The increased cytotoxicity of monodesmosidic vs bidesmosidic saponins is already reported in the literature for quinoa saponins [26,36].

### 2.4. Activity Modulation–In Silico Evaluation of the Mono- vs. Bidesmosidic Saponin Activities

**Figure 13:**
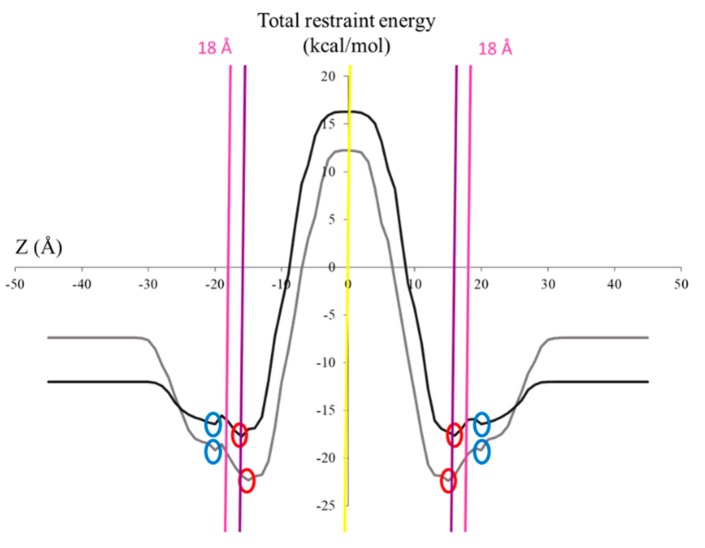
IMPALA simulation of Saponin O (dark line) and Saponin O^h^ (grey line) traversing a 36 Å thick implicit membrane. The “energy-like” profile of the saponin traversing the implicit bilayer. The Z-axis corresponds to the position of the center of mass of the saponin along an axis orthogonal to the membrane, the center of the bilayer corresponding to the intersection with the Y-axis. The different circles represent the two most stable positions of both molecules the center of the bilayer corresponds to the Y-axis with the red circles corresponding to the most stable positions. The vertical lines represent the water/membrane interface (pink), the hydrophilic head/hydrophobic tail interface of the phospholipid bilayer (purple), and the center of the bilayer (yellow).

In order to further understand the nature of the interaction with the cell membrane discussed above, an in silico approach called the IMPALA [37], was applied to analyze the propensity of the saponins to insert into an implicit lipid bilayer mimicking a plasma membrane [18]. For the present simulation, we selected Saponins O and O^h^ that are represented in Figure 1. In Figure 13, the Z-axis represents the position of the mass center of the saponin molecules when orthogonally penetrating in a 36 Å-thick (2 x 18 Å) bilayer membrane. The intercept between the Z-and the Y-axis corresponds to the center of the bilayer membrane. The Y-axis presents the energies of the interaction between the implicit bilayer and the saponin molecule. From Figure 13, we can note that the two studied saponins possess two stable positions (most negative total restraint energies). In addition, the highly positive energies of interaction observed in the hydrophobic center (alkyl chains) of the phospholipid bilayer (±15.75 to ±0 Å) indicate that the amphiphilic nature of both saponins inhibits them from passively crossing the bilayer. The most stable positions for each saponin, highlighted by red circles, are at ± 16 Å with an energy of −17,7 kcal/mol for Saponin O and at ± 15 Å with an energy of −22.4 kcal/mol for Saponin O^h^. For the hydrolyzed saponin, this indicates that the center-of-mass of the saponin is located deeper in the membrane and in particular is now positioned below the hydrophilic/hydrophobic interface of the phospholipid bilayer (± 15.75 Å–purple vertical line in Figure 13). Moreover, the increased penetration of the hydrolyzed molecule into the membrane goes hand in hand with an increased stabilization. This difference in energy indicates an increase stabilization of the hydrolyzed saponin within the membrane. The monodesmosidic Saponin O^h^ has a more favorable interaction than the bidesmosidic, yielding Saponin O^h^ with the more stable position at the polar/apolar interface of the implicit bilayer.

In concordance with hemolytic data, we propose that monodesmosidic molecules have a stronger interaction with membrane phospholipids than bidesmosidic saponins, thus disturbing the membrane to a greater extent. The removal upon hydrolysis of the glucose bonded at C28 creates in the hydrolyzed saponin molecules a more defined separation between the polar and the apolar moieties, allowing for more favorable interactions with membrane phospholipids and thus increasing their hemolytic activity.

## 3. Conclusions

*Chenopodium quinoa* seed is a staple food and a superfood widely used around the world due to high nutritive values and the resistance of the crop to drought, cold and hard climate. However, the husk represents a waste that contains huge amounts of saponins that certainly deserve valorization. In the present study, we quantitatively converted a complete set of bidesmosidic saponins into their monodesmosidic analogs using a fast and green approach based on a microwave assisted hydrolysis in water.The optimized reaction conditions for the hydrolysis was heating at 150ºC, for 5 min while maintaining pH at 10. Using mass spectrometry, we qualitatively and quantitively characterized the natural extract as well as the hydrolysate, and we confirmed the quantitative and selective hydrolysis of the ester linkage in C_28_. The influence of the topology on the membranolytic properties of the saponin molecules, natural bidesmosidic vs generated monodesmosidic ones, was then monitored using the hemolytic activity assay. The cytotoxicity of the hydrolyzed saponins appears significantly increased in agreement with the associated structural modifications. In silico investigations, using the IMPALA in silico procedure, further confirmed that the monodesmosidic saponins interact more preferentially with bilayer phospholipids than bidesmosidic saponins, therefore explaining the increased hemolytic activity that was detected experimentally.

To the best of our knowledge, this study represents the first work that associates the complete structural characterization of chemically modified saponins to their cytotoxic activities. Such a work will pave the way to the valorization of quinoa husks via the ecofriendly modification of the contained saponins. Moreover, the thorough structural characterization performed in the study may afford valuable pieces of information towards the elucidation of the structure-activity relationship of saponins.

## 4. Materials and Methods

### Chemicals, Plant Sampling and Saponin Extractions

#### Chemicals

For saponin extractions, alkaline hydrolysis and mass spectrometry analysis, technical grade methanol, hexane, dichloromethane, chloroform and isobutanol, as well as HPLC grade water, acetonitrile and methanol, were purchased from CHEM-LAB NV (Somme-Leuze, Belgium). N,N-Dimethylaniline (DMA) and 2,5-dihydroxybenzoic acid (DHB) were provided by Sigma-Aldrich (Diegem, Belgium). Disodium phosphate was purchased from Merck (Overijse, Belgium) and sodium hydroxide from VWR.

#### Saponin Extraction from Quinoa Husks

For the quinoa source, integuments of mature achenes were obtained from pooled samples from the Quinoa Breeding Program from Instituto Nacional dé Investigación Agraria (INIA) Chile. To separate kernels from the outer husk, seeds were subjected to physical shearing and the kernels were discarded. The remaining husks have a particle size less than 1 mm in diameter and were immediately submitted to the extraction method adapted from Van Dyck et al. [20]. The weighed powder is stirred in methanol for 24 h at room temperature followed by filtration. The extracts are diluted to 70% methanol with MilliQ water. These methanolic extracts are partitioned (*v*/*v*) successively against n-hexane, dichloromethane and chloroform. Finally, the hydromethanolic solution is evaporated at low pressure in a double boiler at 46 °C using a rotary evaporator. The dry extract is diluted in water to undergo a last partitioning against isobutanol (*v*/*v*). The butanolic phase is washed twice with water to remove salts and impurities. The organic solution contains the saponins and is evaporated to obtain a powder.

#### Microwave Hydrolysis

The hydrolysis is adapted from Sun et al. [26]. Quinoa husk extract-butanolic fraction (4 mg) is solubilized in 4 mL of different buffer solution covering a range of pH values. *pH 7*: 50mL of KH_2_PO_4_ 0.1 mol/L is added to 29.1 mL of NaOH 0.1mol/L and brought to 100 mL with Milli-Q water. *pH 8*: 50 mL of KH_2_PO_4_ 0.1 mol/L is added to 46.7 mL of NaOH 0.1 mol/L and brought to 100 mL with Milli-Q water. *pH 9*: 50 mL of borax 0.025 mol/L is added to 4.6 mL of HCl 0.1 mol/L and are brought to 100 mL with Milli-Q water. *pH 10*: 50 mL of borax 0.025 0.1mol/L is added to 18.3 mL of NaOH 0.1 mol/L and brought to 100 mL with Milli-Q water. *pH 11*: 50 mL of Na_2_HPO_4_ 0.05 mol/L is added to 4.1 mL of NaOH 0.1 mol/L and brought to 100 mL with Milli-Q water. *pH 12*: 50 mL of Na_2_HPO_4_ 0.05 mol/L is added to 26.9 mL NaOH 0.1 mol/L and brought to 100 mL with Milli-Q water. *pH 13*: 0.4 g of NaOH is dissolved in 100 mL Milli-Q water to reach 0.1 mol/L. *pH 14*: 4 g of NaOH is dissolved in 100 mL Milli-Q water to reach 1 mol/L. The sample is heated at 150 °C for 5 min using an Initiator Classic microwave setup (Biotage^®^, Uppsala, Sweeden). After cooling at R.T., the pH of the solution is adjusted to 7 with hydrochloric acid (0.1%). The aqueous solution is extracted with isobutanol (*v*/*v*). The organic phase is recovered and washed twice with water to remove salts. The organic layer is finally evaporated under reduced pressure to obtain a white solid (50% yield).

#### Mass Spectrometry Analysis

We developed a mass spectrometry protocol based on MALDI-HRMS, LC-MS, LC-MSMS and LC-IMS analysis to perform a thorough saponin characterization [23,24]. The saponin extract from butanolic fraction is first analyzed using *MALDI-MS* on a Q-TOF Premier mass spectrometer (Waters, Manchester, UK) in the positive ion mode. The MALDI source is constituted of a Nd-YAG laser, operating at 355 nm, with a maximum pulse energy of 104.1 μJ delivered in 2.2 ns to the sample at 200 Hz repeating rate. All samples contained the matrix, a mixture of 25 mg of DHB in 250 μL water/acetonitrile (*v*/*v*) with 6 μL of DMA. The dried-droplet method is selected to prepare the sample/matrix co-crystal on the target plate. In this method, the saponin extract is not premixed with the matrix. A sample droplet (1 μL) is applied on top of a fast-evaporated matrix-only bed. For the recording of the single-stage MALDI-MS spectra, the quadrupole (rf-only mode) is set to pass ions between *m*/*z* 250 and 2000 and all ions are transmitted into the pusher region of the TOF analyzer where they are mass-analyzed with an 1s integration time. Accurate mass measurements are also performed using MALDI with PEG600-1500 as the external standard (lock masses). This first step allows to validate the presence of saponin congeners based on the measure of their elemental composition (Table 1).

#### LC-MS Analyses

These are performed with a Waters Acquity H-class liquid chromatography device coupled to a Waters Synapt G2-Si mass spectrometer. The HPLC part consists of a vacuum degasser, a quaternary pump and an autosampler. Sample volumes of 1 µL are injected. Chromatographic separation is performed on a non-polar column (Acquity UPLC BEH C18; 2.1 × 50 mm; 1.7 µm; Waters) at 40 °C. The mobile phase is programmed with a constant flow (0.25 mL/min) and consists of an elution gradient starting with 85 % of eluent A (water, 0.1 % formic acid) and 15 % of eluent B (acetonitrile) and reaching 60 % of eluent A and 40 % eluent B at 6 min. This ratio is maintained for 3 min, then modified again to reach 5 % eluent A and 95 % eluent B at 11 min. This second ratio is maintained for 1 min and, finally, brought back to 85 % eluent A and 15 % eluent B at the end of the chromatographic run (13 min). For the mass spectrometer parameters, the electrospray ionization (ESI) conditions are the same regardless of the ion mode (positive or negative mode); capillary voltage 2.5 kV; cone voltage 40 V; source Offset 80 V; source temperature 100 °C; desolvation temperature 300 °C. Dry nitrogen is used as the ESI gas with a flow rate of 500 l/h for the desolvation gas. For the LC-MS analysis, the quadrupole is set to pass ions from *m*/*z* 50 to 2000 and all ions are transmitted into the pusher region of the time-of-flight analyzer for mass-analysis with 1 s integration time.

Ion mobility experiments are performed using the TWIMS cell constituting the so-called Tri-Wave setup, composed of three successive T-wave elements named the Trap cell, the IMS cell, and the Transfer cell, in which the wave speed and amplitude are user-tunable. The trap and transfer cells are filled with argon whereas the IMS cell is filled with nitrogen. A small rf-only cell filled with helium is fitted between the trap and the IMS cell. This mass spectrometer is used for the recording of the ESI full scan mass spectrum, for the collision-induced dissociation (CID) as well as for the ion mobility experiments. Ion mobility parameters are tuned to have the highest separation between different ion structures. The IMS cell conditions are gas flow 110 mL/min, wave velocity 400 m/s and wave height 40 V. TWIMS data are analyzed using the Waters MassLynx SCN 901 software. Arrival time distributions (ATDs) are recorded by selecting the most abundant isotope for each ion composition to avoid unspecific selection. ATDs are converted into collisional cross-section (CCS) distributions in helium by means of a polymer calibration following a procedure detailed in the literature using commercial PEG samples with average molecular weights of 600 and 1000 g.mol^−1^. [31] The CCSs are determined at the APEX of the CCS distributions.

#### Semi-Quantitative Analysis

As already discussed in previous publications [23], absolute quantification of saponins in an extract would require the availability of samples of each purified saponin as an external standard. This is to date impossible and we will only use relative quantification: (i) to determine the mass fraction of saponins in the quinoa extract (QE) (see Table 1) and (ii) to estimate the relative abundances of each saponin within the extract. This will be achieved by spiking a known amount of Hederacoside C (Sigma-Aldrich), a commercially available saponin purified from *Hedera helix,* as the internal standard. [4] The integration of the LC-MS signals will then allow estimating the concentration–relative to hederacoside C - of each saponin in the extract. The total saponin mass in the quinoa husk extract is further compared to the mass of the quinoa husk powder submitted to the extraction procedure (see Table 1 and Table 2) to estimate the saponin content contained in the quinoa husk.

#### Hemolytic Activity

The hemolytic activity is determined on bovine red blood cells. Fresh blood samples are collected in a citrate solution (0.11 mol/L) and stored in a fridge (4 °C). The erythrocyte lysis causes the release of hemoglobin and heme. The hemolytic activity is based on the determination of the heme concentration. In a 50mL vial, 10 mL of citrated are added to 40 mL of phosphate buffer saline (PBS). The sample is centrifuged (1000× *g*, 10 min) in a Heraeuos Biofuge Stratos centrifuge (Waltham, MA, USA) to wash the erythrocytes and the supernatant is removed until it becomes colorless. The pellet is conserved and diluted in PBS to reach a concentration of 2 % (2 mL erythrocyte in 98 mL of PBS). At the same time, solutions containing saponin extract are prepared at different concentrations. After that, 15 µL of extract solutions are placed in 1485 µL of erythrocyte suspension, each sample is carried out in triplicates. The final concentration is formulated in µg of extract per mL of 2 % erythrocyte suspension at 0.5/1/2/3/4/5/10/20/30/40/50/100/200/300. Due to the poor water solubility of hydrolyzed quinoa, the saponin concentration cannot exceed 300 µg·mL^−1^. The solutions are shaken in the incubator (T-Mix, Analytik Jena, Endress Houser Co., Jena, Germany) during 1 h at 500 rpm to allow the interaction between saponins and erythrocytes. After that, samples are centrifuged (1000× *g*, 10 min). The supernatant containing heme of each sample is retrieved and 100 µL is placed in a 96-well plate. Finally, the absorbance of each well is read at 540 nm in spectrophotometer (which one) and subtracted by the absorbance of a blank (saponin-free solution) to determine the hemolytic activity.

#### In silico Study

The most probable 3D conformations of the saponins of interest are first determined by considering the main torsional angles of the molecules and intramolecular energies of interaction, using an empirical force field previously described [18,37]. Using these 3D structures, the in silico IMPALA technique as first described by Ducarme et al. [37] is used to assess the extent to which an amphiphilic molecule is likely to penetrate a model phospholipid bilayer. The IMPALA method uses a membrane model in which the membrane bilayer is implicitly modeled by an empirical function C(z), see Equation 1. This model assumes that the properties of the implicit membrane are constant in the X,Y plane and only vary along the perpendicular Z (in Å) axis that originates at the bilayer center. C(z) varies from 1 (completely hydrophilic) to 0 (completely hydrophobic):

(1) $$C\left(z\right)=1-\frac{1}{1+e^{\propto \left(\left|z\right|-z_{0}\right)}}\;$$

where α is a constant equal to 1.99, z_0_ is the position of the hydrophilic/hydrophobic interface in the membrane and z is the position in the membrane. The total thickness of the bilayer was set at 36 Å.

The IMPALA method uses two energy restraints [18], a hydrophobic restraint and a lipid perturbation restraint, to simulate the interactions between the molecule of interest and the lipid bilayer. The saponin is translated across the implicit bilayer along the Z axis 1 Å at a time and rotated 360° at each position z(i), and the sum of the two restraints is calculated to predict the most stable position (with the lowest energy) into the implicit membrane.

## Figures and Tables

**Table 1:** Chenopodium quinoa natural extract (see Figure 2 for the general saponin structure): identification by MS-based methods of the saponin content. Hed, SA, OA and PA stand respectively for hederagin, serjanic acid, oleanic acid, and phytolaccagenic acid. AG489 corresponds to the aglycone #489 as defined in [6] and presented in Figure 3. RT stands for Retention Time in LC-MS experiments. CCS correspond to collision cross sections determined using traveling wave (TW) ion mobility with Nitrogen as the buffer gas and converted in CCS against Helium using a calibration procedure [31], namely ^TW^CCS_N2⭢He_ using the newly introduced nomenclature [33]. The Molar Proportion (% in the quinoa husk extract) and the mass fraction (mg·g^−1^ of the quinoa husk powder) of each saponin is estimated based on the ion signal ratios as determined by mass spectrometry experiments (LC-MS), with hederacoside C as an external standard.

	Composition	*m*/*z* [M + Na]^+^	Δ(*m*/*z*) (ppm)	R_1_	R_2_	Aglycone	R_3_	RT (min)	CCS (Å²) [M + Na]^+^	CCS (Å²) [M + H]^+^	Molar Proportion(%)	Mass Fraction (mg·g^−1^)
I	C_47_H_76_O_18_	951.4929	2.1	- CH_3_	- CH_2_OH	Hed	Glc – Ara -	7.3	234	254	14.9	6.1
Unknown-1	C_47_H_74_O_19_	965.4722	0.2	?	?	?	Glc – Ara -	5.3	235	254	1.9	0.8
19	C_47_H_76_O_19_	967.4878	4.9	- CH_2_OH	- CH_2_OH	AG489	Xyl - Glc -	4.0	238	254	1.6	0.7
19a				- CH_2_OH	- CH_2_OH	AG489	Xyl - Glc -	4.8	238	256	0.3	0.1
H	C_48_H_76_O_19_	979.4878	1.5	- COOCH_3_	- CH_3_	SA	Glc – Ara -	7.1	235	NA	1.6	0.7
70				- CH_3_	- CH_3_	OA	Glc – GlcA -	7.5	235	NA	0.9	0.4
Q	C_48_H_78_O_19_	981,5035	0.8	- CH_3_	- CH_2_OH	Hed	Glc – Gal -	6.5	235	260	0.2	0.1
B	C_48_H_76_O_20_	995.4828	3.2	- COOCH_3_	- CH_2_OH	PA	Glc – Ara -	4.9	243	260	61.7	25.3
61	C_53_H_86_O_23_	1113.5458	1.6	- CH_3_	- CH_2_OH	Hed	Glc – Glc – Ara -	5.9	275	290	0.7	0.3
Unknown-2	C_53_H_86_O_24_	1127.5251	2.1	?	?	?	Glc – Glc – Ara -	4.5	270	290	1.7	0.7
G	C_54_H_86_O_24_	1141.5407	3.6	- COOCH_3_	- CH_3_	SA	Glc – Glc – Ara -	5.7	266	291	3.0	4.6

**Table 2:** Microwave-assisted alkaline hydrolysis (5min, pH 10, 150 °C) of Chenopodium quinoa saponin extract: identification by MS-based methods of the saponin content. Hed, SA, OA and PA stand respectively for hederagin, serjanic acid, oleanic acid, and phytolaccagenic acid. AG489 corresponds to the aglycone #489 as defined in [6] and presented in Figure 3. RT stands for Retention Time in LC-MS experiments. CCS correspond to collision cross sections determined using traveling wave (TW) ion mobility with nitrogen as the buffer gas and converted in CCS against Helium using a calibration procedure [31], namely ^TW^CCS_N2⭢He_ using the newly introduced nomenclature [33]. The Molar Proportion (% in the hydrolyzed quinoa husk extract) of each saponin is estimated based on the ion signal ratios as determined by mass spectrometry experiments (LC-MS), with hederacoside C as an external standard.

	Composition	*m*/*z* [M + Na]^+^	Δ(*m*/*z*) (ppm)	R_1_	R_2_	Aglycone	R_3_	RT (min)	CCS (Å²) [M + Na]^+^	CCS (Å²) [M + H]^+^	Molar Proportion (%)
**I^h^**	C_41_H_66_O_13_	789.4401	0.8	- CH_3_	- CH_2_OH	Hed	Glc – Ara -	10.7	224	219	16.5
**Unknown-1^h^**	C_41_H_64_O_14_	803.4194	0.4	?	?	?	Glc – Ara -	7.2	225	221	2.4
**19^h^**	C_41_H_66_O_14_	805.4350	0.7	- CH_2_OH	- CH_2_OH	AG489	Xyl - Glc -	5.6	227	221	2.2
**19a^h^**				- CH_2_OH	- CH_2_OH	AG489	Xyl - Glc -	5.8	NA	NA	0.1
**H^h^**	C_42_H_66_O_14_	817.4350	2.1	- COOCH_3_	- CH_3_	SA	Glc – Ara -	9.4	240	NA	2.2
**70^h^**				- CH_3_	- CH_3_	OA	Glc – GlcA -	13.1	235	NA	1.0
**Q^h^**	C_42_H_68_O_14_	819.4501	0.6	- CH_3_	- CH_2_OH	Hed	Glc – Gal -	9.4	228	222	0.1
**B^h^**	C_42_H_66_O_15_	833.4299	1.1	- COOCH_3_	- CH_2_OH	PA	Glc – Ara -	9.9	234	227	61.4
**61^h^**	C_47_H_76_O_18_	951.4929	1.3	- CH_3_	- CH_2_OH	Hed	Glc – Glc – Ara -	10.2	250	253	0.4
**Unknown-2^h^**	C_47_H_74_O_19_	965.4722	2.5	?	?	?	Glc – Glc – Ara -	7.1	252	254	0.9
**G^h^**	C_48_H_76_O_19_	979.4879	0.5	- COOCH_3_	- CH_3_	SA	Glc – Glc – Ara -	9.4	265	NA	2.7
**O^h^**	C_48_H_76_O_20_	995.4828	0.4	- COOCH_3_	- CH_2_OH	PA	Glc – Glc – Ara -	7.6	258	263	10.1

## Supplementary Materials

The following are available online at https://www.mdpi.com/1420-3049/25/7/1731/s1: Figures S1–24: LC-MSMS analysis of Chenopodium quinoa husk saponin extract: CID spectra recorded for all the saponin ions from Table 1 and Table 2; Table S1: Hemolytic activity assay of the hydrolyzed and natural extract saponins. Average and standard deviation of the free heme absorbance (540 nm) with regards to the increasing saponin concentration.
