# Supplementary material for: Enhancing the Membranolytic Activity of Chenopodium quinoa Saponins by Fast Microwave Hydrolysis
Source: Molecules. 2020 Apr 9;25(7):1731. doi: 10.3390/molecules25071731 (PMC7181122; doi:10.3390/molecules25071731)
Supplement: Supplementary file 1 [file molecules-25-01731-s001.pdf]

Supplementary Information

Enhancing the Membranolytic Activity of *Chenopodium quinoa* Saponins by Fast Microwave Hydrolysis

Emmanuel Colson [1,2], Philippe Savarino [1], Emily J.S. Claereboudt [2,3], Gustavo Cabrera-Barjas [4], Magali Deleu [3], Laurence Lins [3], Igor Eeckhaut [2], Patrick Flammang [2] and Pascal Gerbaux [1,*]

[1] Organic Synthesis and Mass Spectrometry Laboratory (S²MOs), University of Mons, 23 Place du Parc, 7000 Mons, Belgium; emmanuel.colson@umons.ac.be (E.C.); Philippe.savarino@umons.ac.be (P.S.)

[2] Biology of Marine Organisms and Biomimetics Unit (BOMB), University of Mons, 23 Place du Parc, 7000 Mons, Belgium; Emily.CLAEREBOUDT@umons.ac.be (E.J.S.C.); igor.eeckhaut@umons.ac.be (I.E.); Patrick.Flammang@umons.ac.be (P.F.)

[3] Laboratory of Molecular Biophysics at Interfaces, TERRA Research Center, Gembloux Agro-Bio Tech, University of Liege, 5030 Gembloux, Belgium; magali.deleu@uliege.be (M.D.); l.lins@ulg.ac.be (L.L.)

[4] Universidad de Concepción, Unidad de Desarrollo Tecnológico (UDT), Av. Cordillera 2634, Parque Industrial Coronel, P.O. Box 4051 mail 3, Coronel, Región del BíoBío, Chile; g.cabrera@udt.cl (C.C.-B.)

* Correspondence: pascal.gerbaux@umons.ac.be

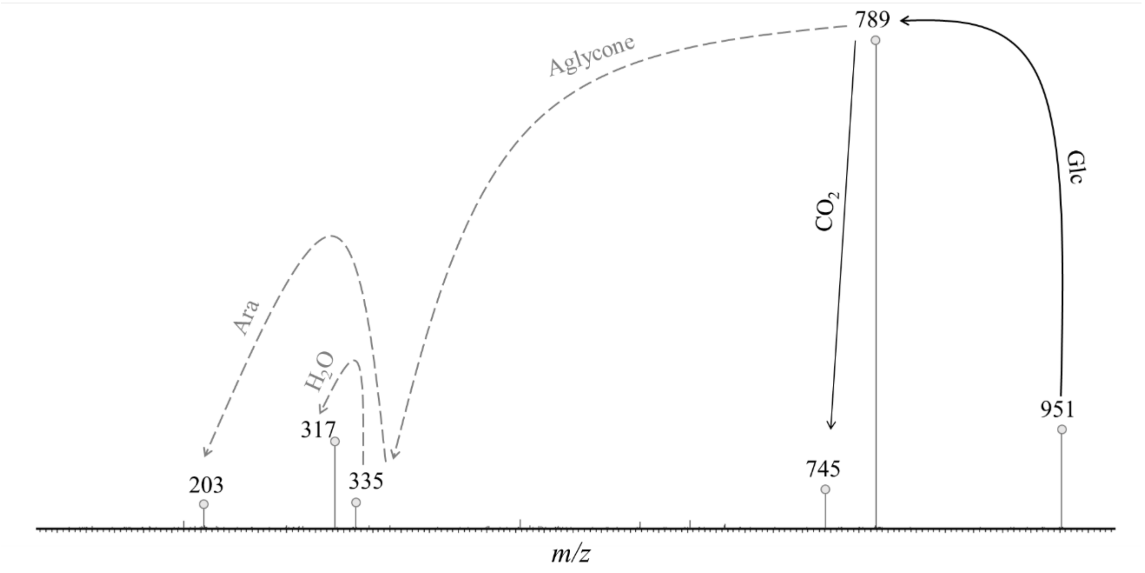

Figure S1a: LC-MSMS analysis of *Chenopodium quinoa* husk saponin extract: CID spectrum recorded for the *m*/*z* 951 precursor ions at 7.3 min retention time. The corresponding ions are assigned as $[M+Na]^+$ ions from Saponin I.

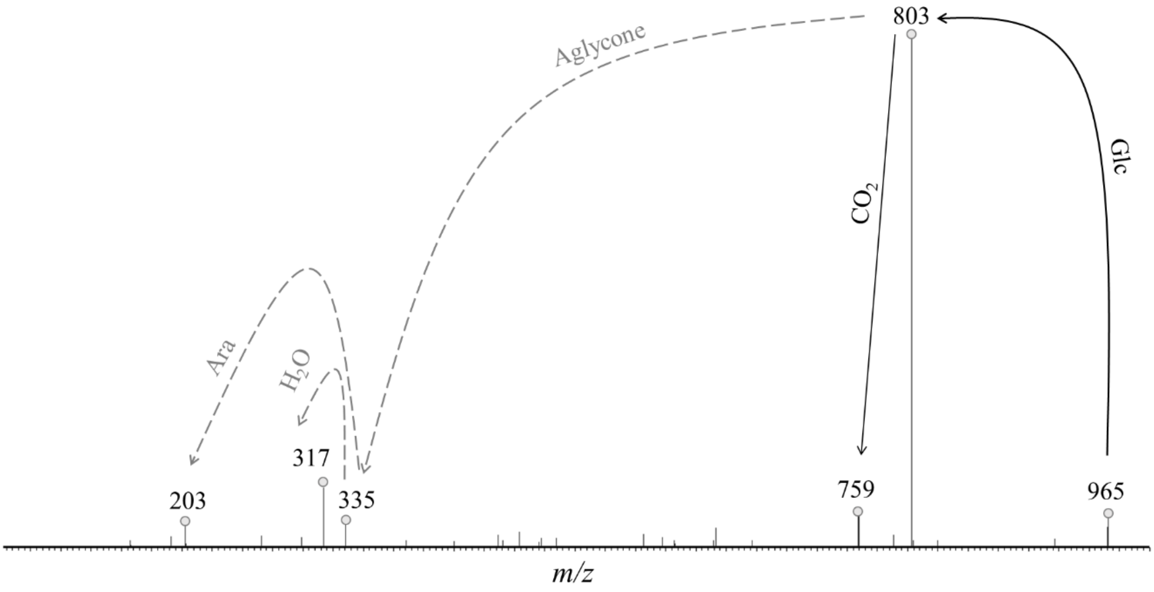

Figure S1b. LC-MSMS analysis of Chenopodium quinoa husk saponin extract: CID spectrum recorded for the m/z 965 precursor ions at 5.3 min retention time. The corresponding ions are assigned as $[M+Na]^+$ ions from Saponin ?.

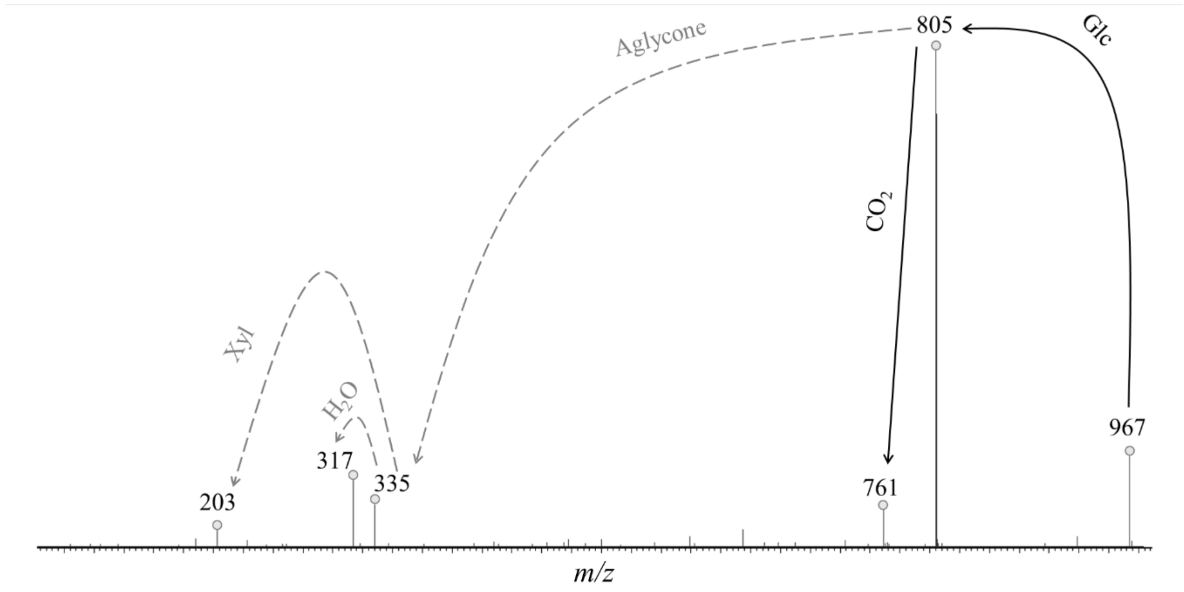

Figure S1c. LC-MSMS analysis of Chenopodium quinoa husk saponin extract: CID spectrum recorded for the m/z 967 precursor ions at 4 min retention time. The corresponding ions are assigned as [M+Na]+ ions from Saponin 19.

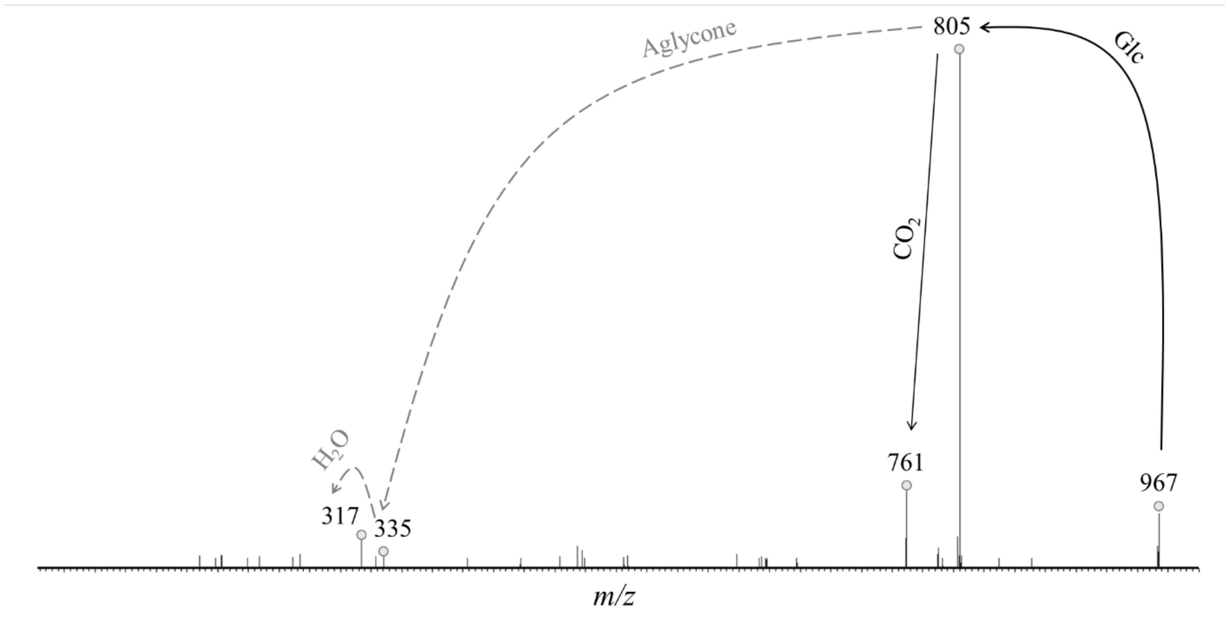

Figure S1d. LC-MSMS analysis of Chenopodium quinoa husk saponin extract: CID spectrum recorded for the m/z 967 precursor ions at 4.8 min retention time. The corresponding ions are assigned as [M+Na]+ ions from Saponin 19a.

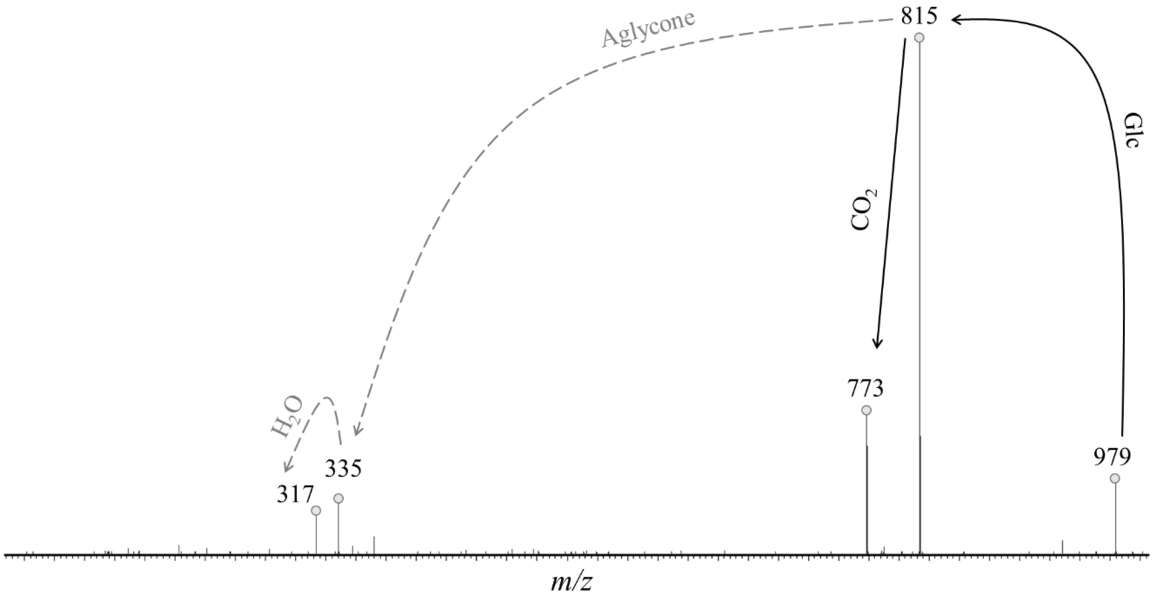

Figure S1e. LC-MSMS analysis of *Chenopodium quinoa* husk saponin extract: CID spectrum recorded for the *m/z* 979 precursor ions at 7.1 min retention time. The corresponding ions are assigned as [M+Na]$^+$ ions from Saponin H.

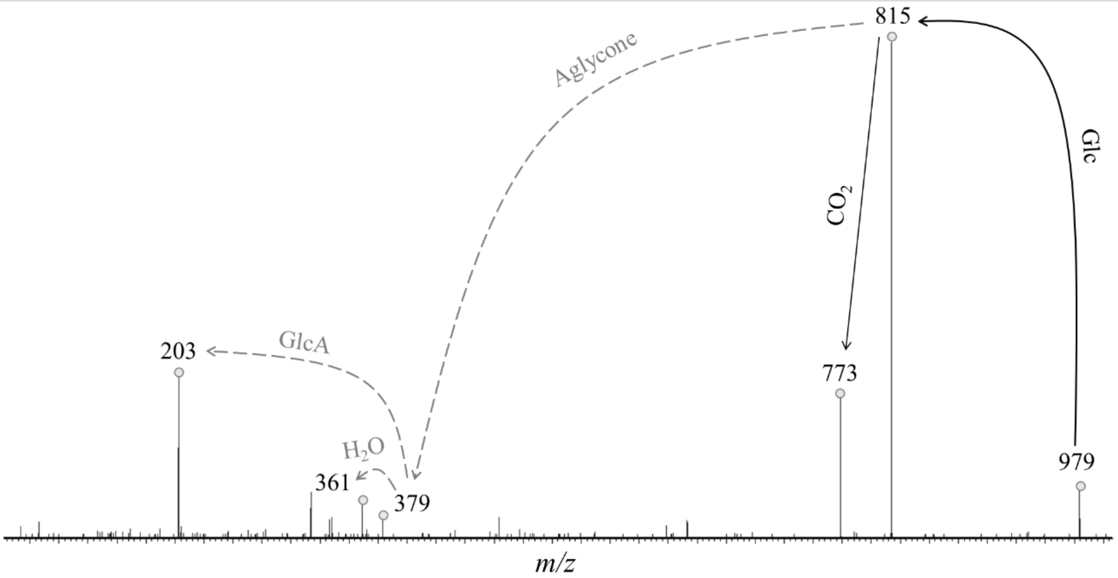

Figure S1f. LC-MSMS analysis of *Chenopodium quinoa* husk saponin extract: CID spectrum recorded for the *m/z* 979 precursor ions at 7.5 min retention time. The corresponding ions are assigned as [M+Na]$^+$ ions from Saponin 70.

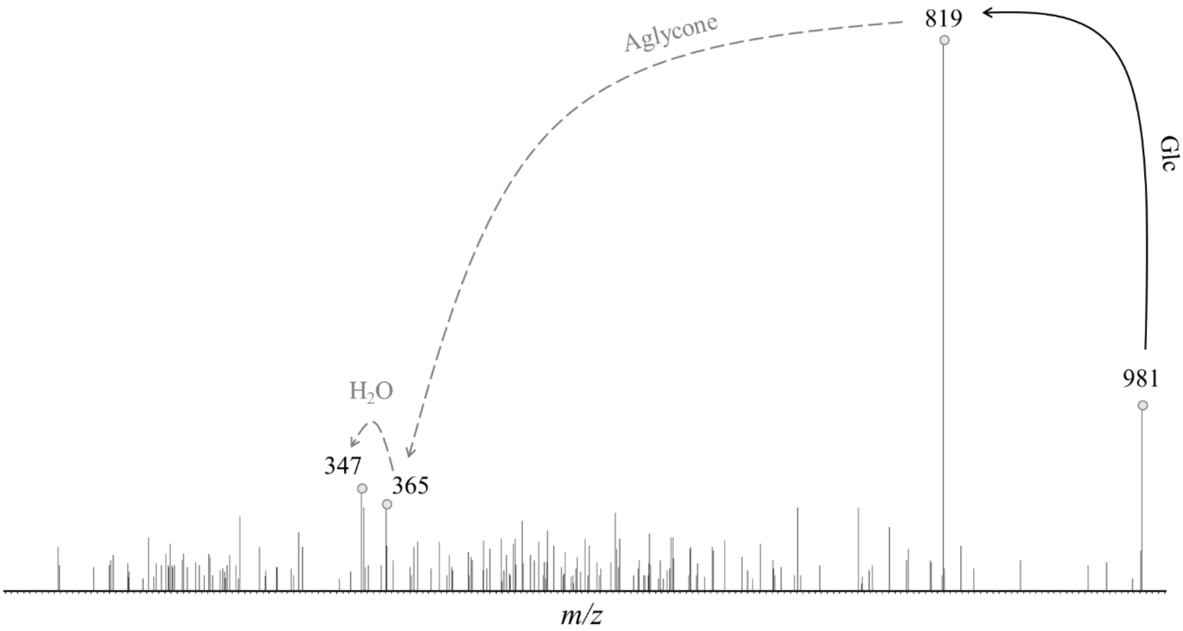

Figure S1g. LC-MSMS analysis of the *Chenopodium quinoa* husk saponin extract: CID spectrum recorded for the *m/z* 981 precursor ions at 6.5 min retention time. The corresponding ions are assigned as $[M+Na]^+$ ions from Saponin Q.

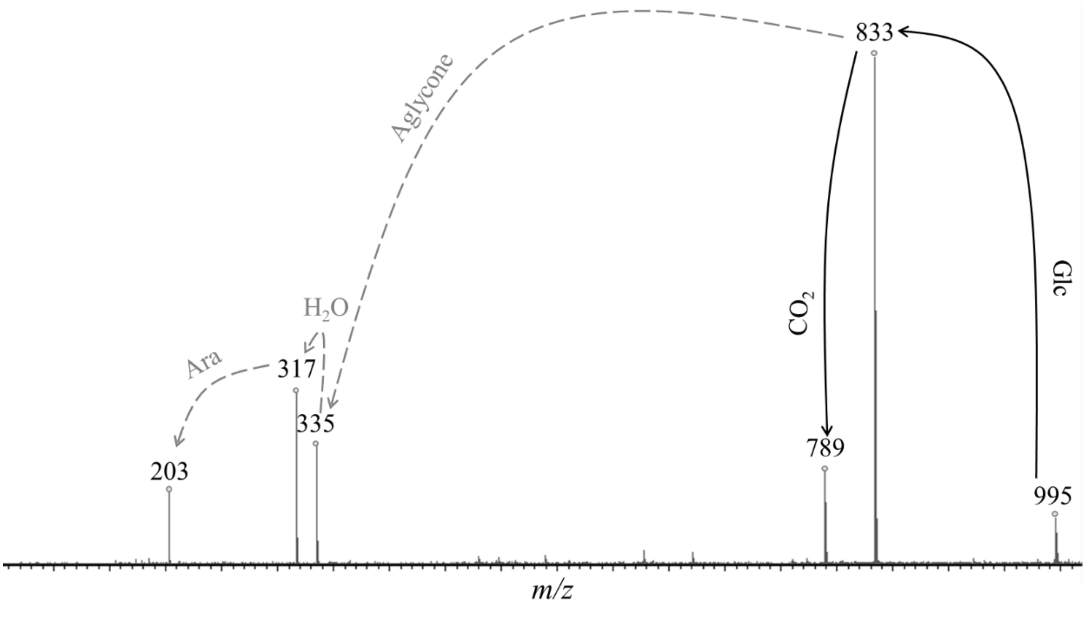

Figure S1h. LC-MSMS analysis of *Chenopodium quinoa* husk saponin extract: CID spectrum recorded for the *m/z* 995 precursor ions at 5.9 min retention time. The corresponding ions are assigned as $[M+Na]^+$ ions from Saponin B.

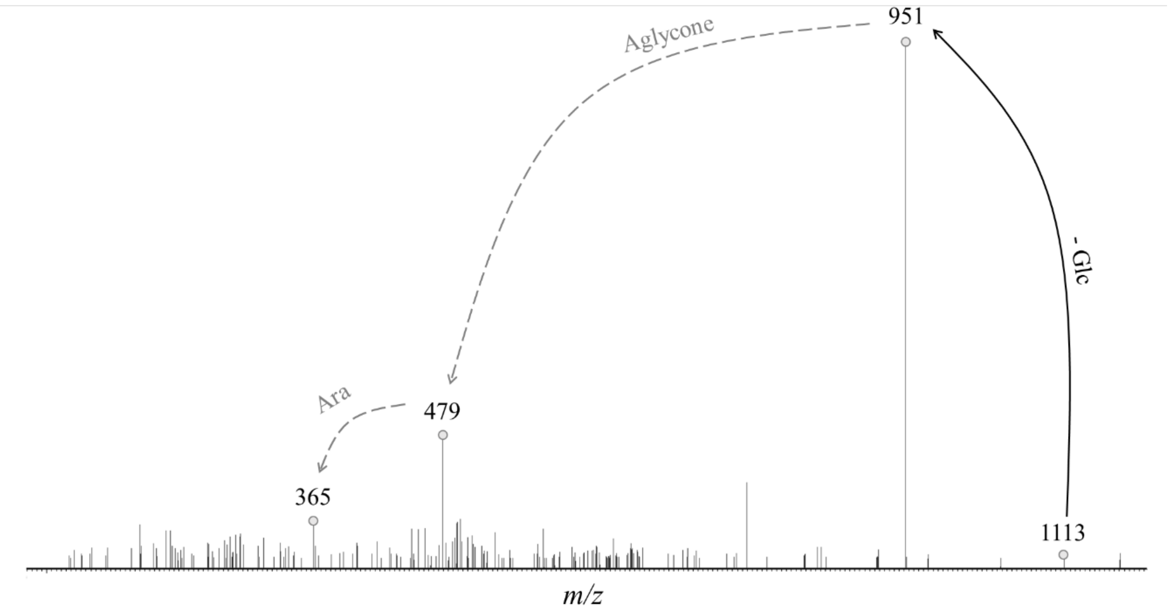

Figure S1i. LC-MSMS analysis of *Chenopodium quinoa* husk saponin extract: CID spectrum recorded for the *m/z* 113 precursor ions at 5.9 min retention time. The corresponding ions are assigned as $[M+Na]^+$ ions from Saponin 61.

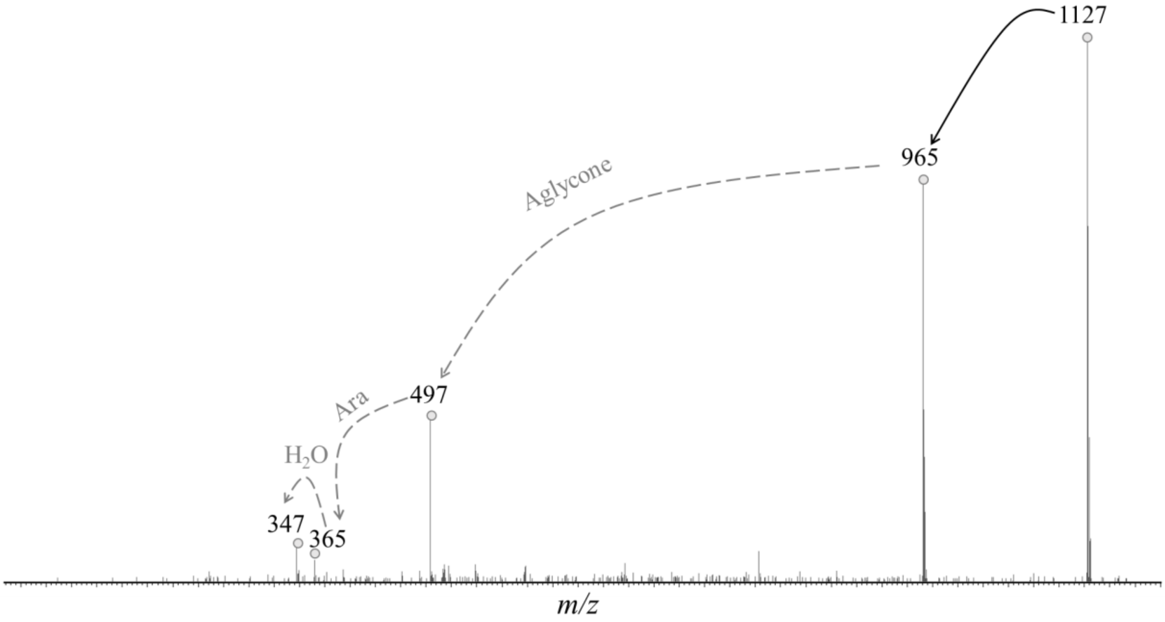

Figure S1j. LC-MSMS analysis of *Chenopodium quinoa* husk saponin extract: CID spectrum recorded for the m/z 1127 precursor ions at 4.5 min retention time. The corresponding ions are assigned as $[M+Na]^+$ ions from Saponin ??.

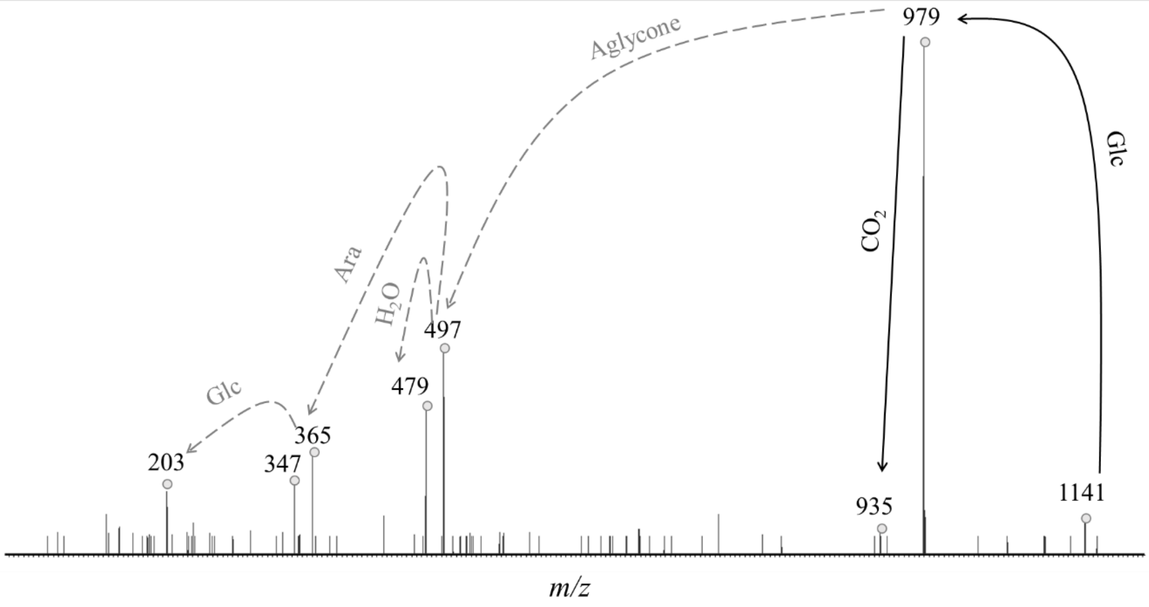

Figure S1k. LC-MSMS analysis of *Chenopodium quinoa* husk saponin extract: CID spectrum recorded for the *m/z* 1141 precursor ions at 5.7 min retention time. The corresponding ions are assigned as $[M+Na]^+$ ions from Saponin G.

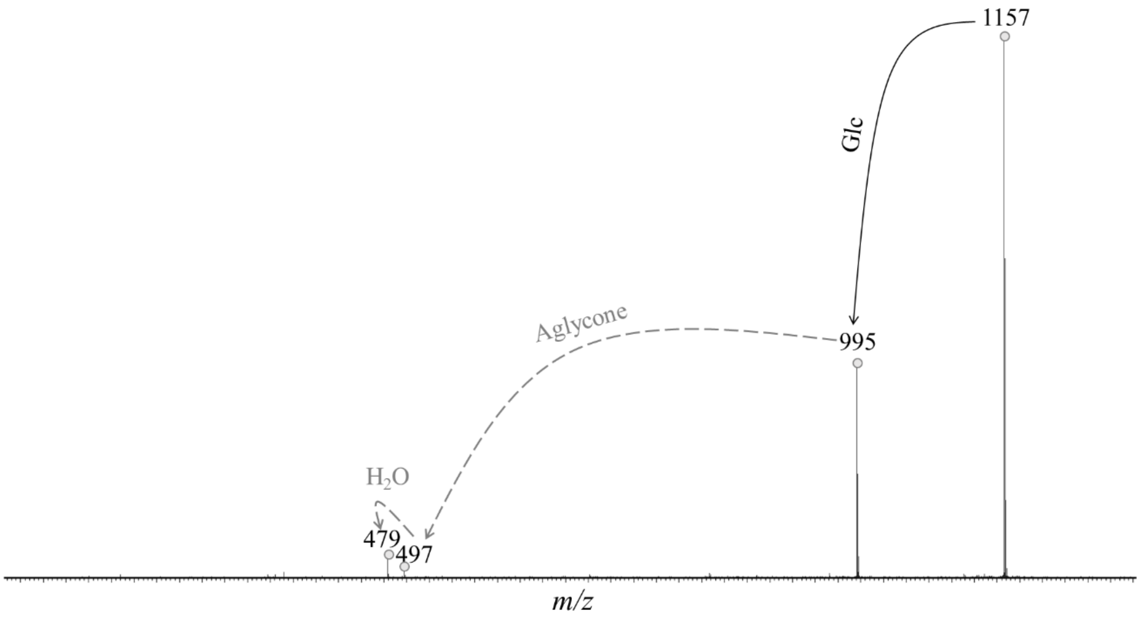

Figure S1l. LC-MSMS analysis of *Chenopodium quinoa* husk saponin extract: CID spectrum recorded for the *m/z* 1157 precursor ions at 4.9 min retention time. The corresponding ions are assigned as $[M+Na]^+$ ions from Saponin O.

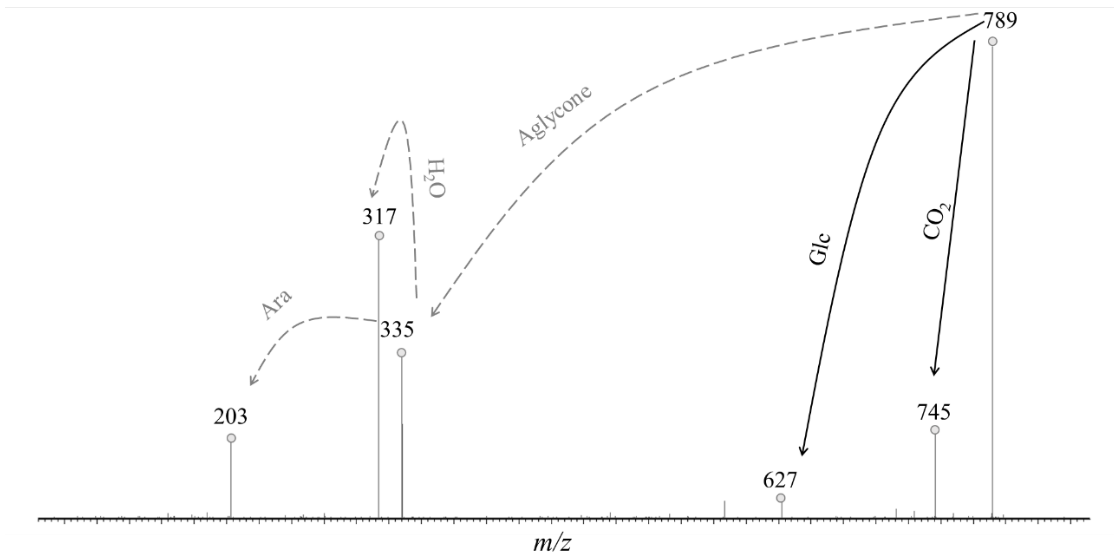

Figure S1m: LC-MSMS analysis of *Chenopodium quinoa* husk saponin extract: CID spectrum recorded for the *m/z* 789 precursor ions at 10.7 min retention time. The corresponding ions are assigned as [M+Na]+ ions from Saponin I[h].

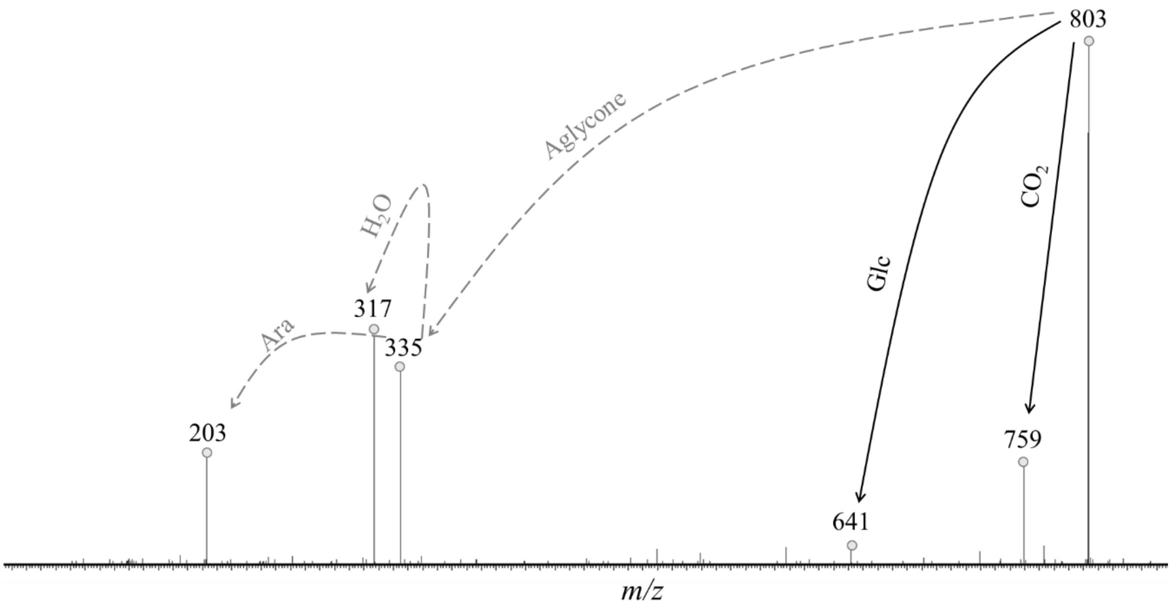

Figure S1n: LC-MSMS analysis of *Chenopodium quinoa* husk saponin extract: CID spectrum recorded for the *m/z* 803 precursor ions at 7.3 min retention time. The corresponding ions are assigned as [M+Na]+ ions from Saponin ?[h].

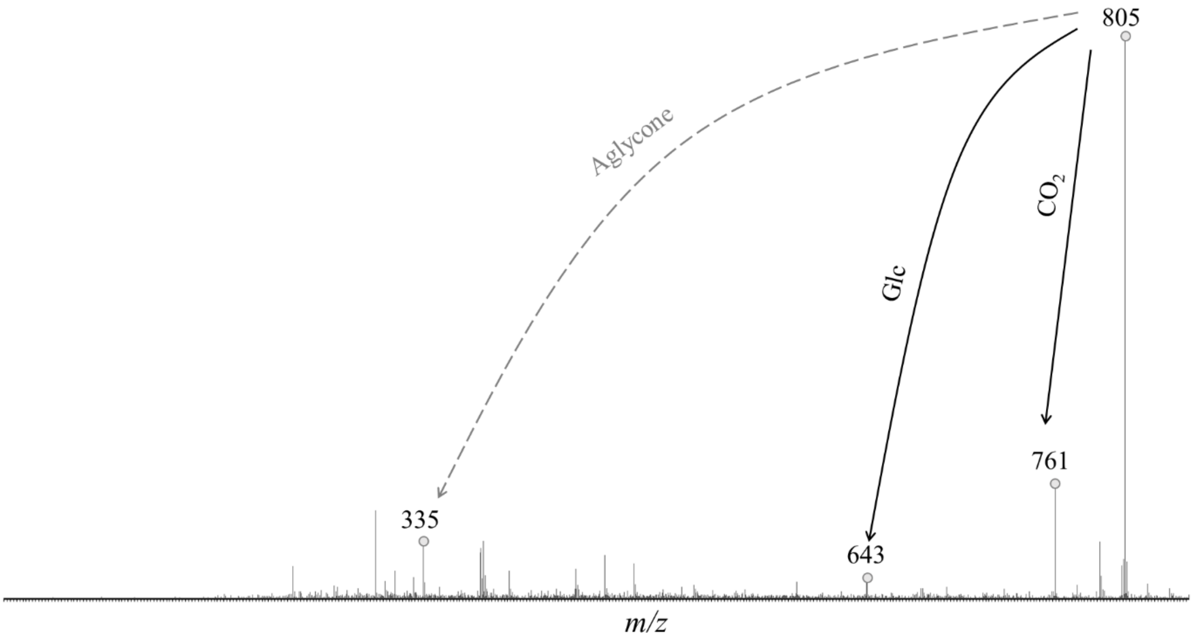

Figure S1o: LC-MSMS analysis of *Chenopodium quinoa* husk saponin extract: CID spectrum recorded for the *m/z* 805 precursor ions at 5.6 min retention time. The corresponding ions are assigned as [M+Na]$^+$ ions from Saponin 19[h].

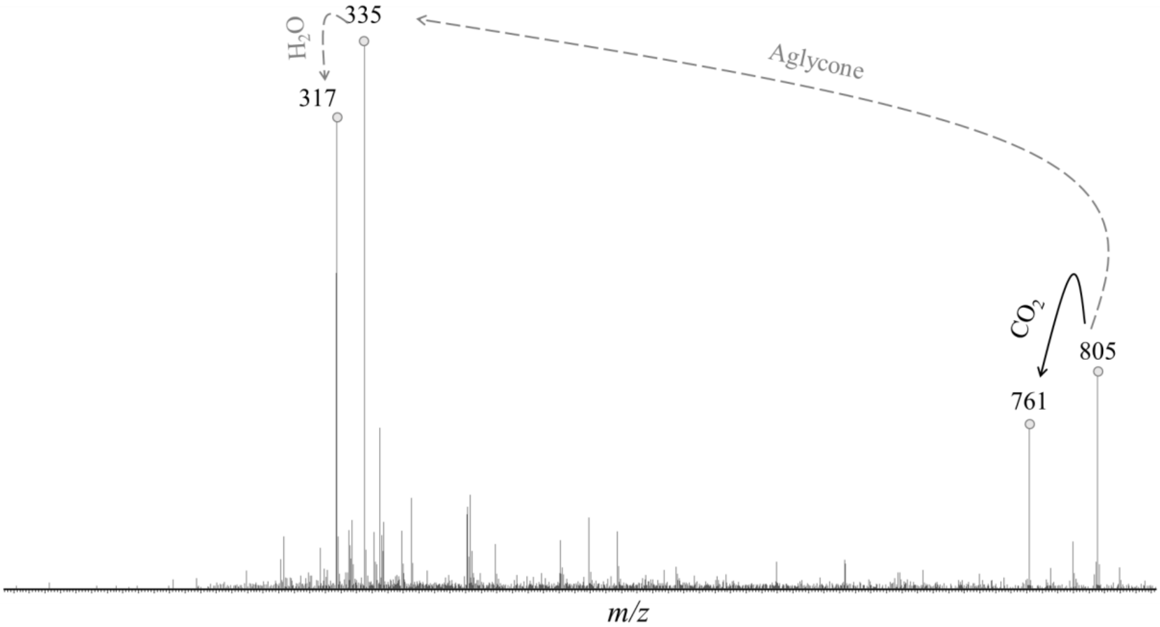

Figure S1p: LC-MSMS analysis of *Chenopodium quinoa* husk saponin extract: CID spectrum recorded for the *m/z* 805 precursor ions at 5.8 min retention time. The corresponding ions are assigned as [M+Na]$^+$ ions from Saponin 19a[h].

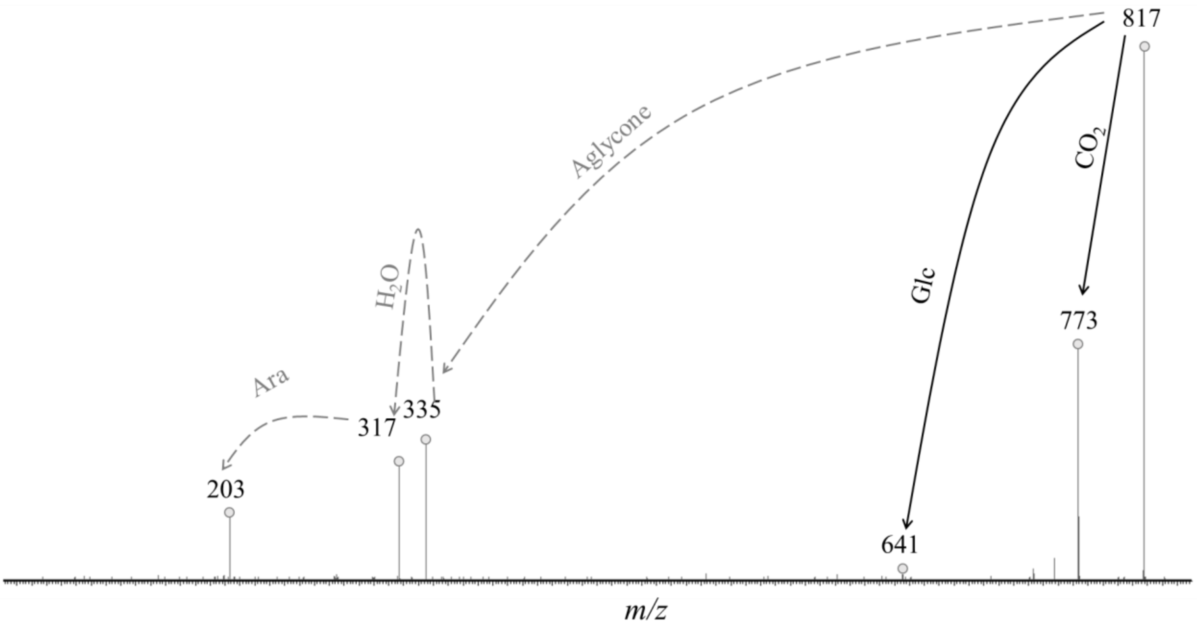

Figure S1q: LC-MSMS analysis *Chenopodium quinoa* husk saponin extract: CID spectrum recorded for the *m/z* 817 precursor ions at 9.4 min retention time. The corresponding ions are assigned as $[M+Na]^+$ ions from Saponin H[h].

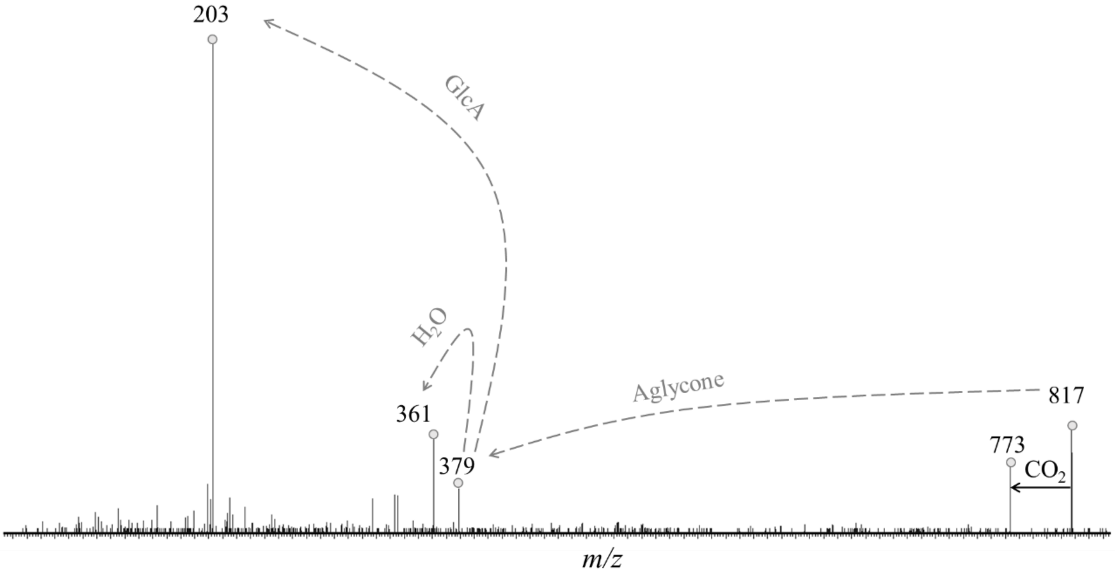

Figure S1r: LC-MSMS analysis of *Chenopodium quinoa* husk saponin extract: CID spectrum recorded for the *m/z* 817 precursor ions at 13.1 min retention time. The corresponding ions are assigned as $[M+Na]^+$ ions from Saponin 70[h].

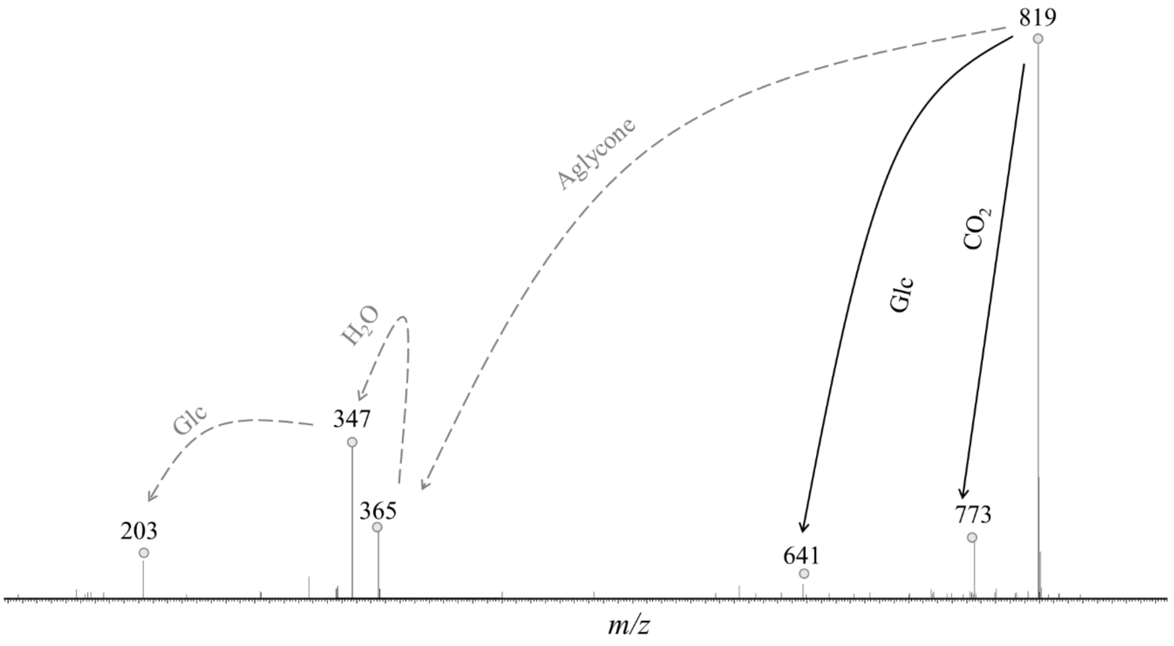

Figure S1s: LC-MSMS analysis of *Chenopodium quinoa* husk saponin extract: CID spectrum recorded for the *m*/*z* 819 precursor ions at 9.4 min retention time. The corresponding ions are assigned as $[M+Na]^+$ ions from Saponin Q^h.

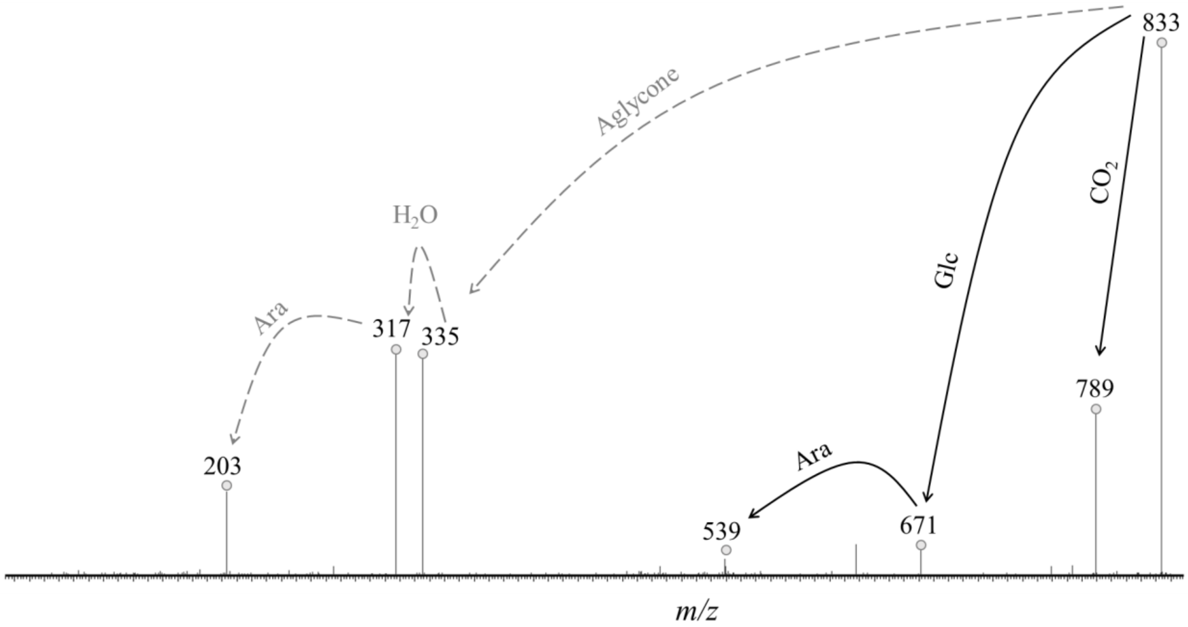

Figure S1t: LC-MSMS analysis of *Chenopodium quinoa* husk saponin extract: CID spectrum recorded for the *m*/*z* 833 precursor ions at 9.9 min retention time. The corresponding ions are assigned as $[M+Na]^+$ ions from Saponin B^h.

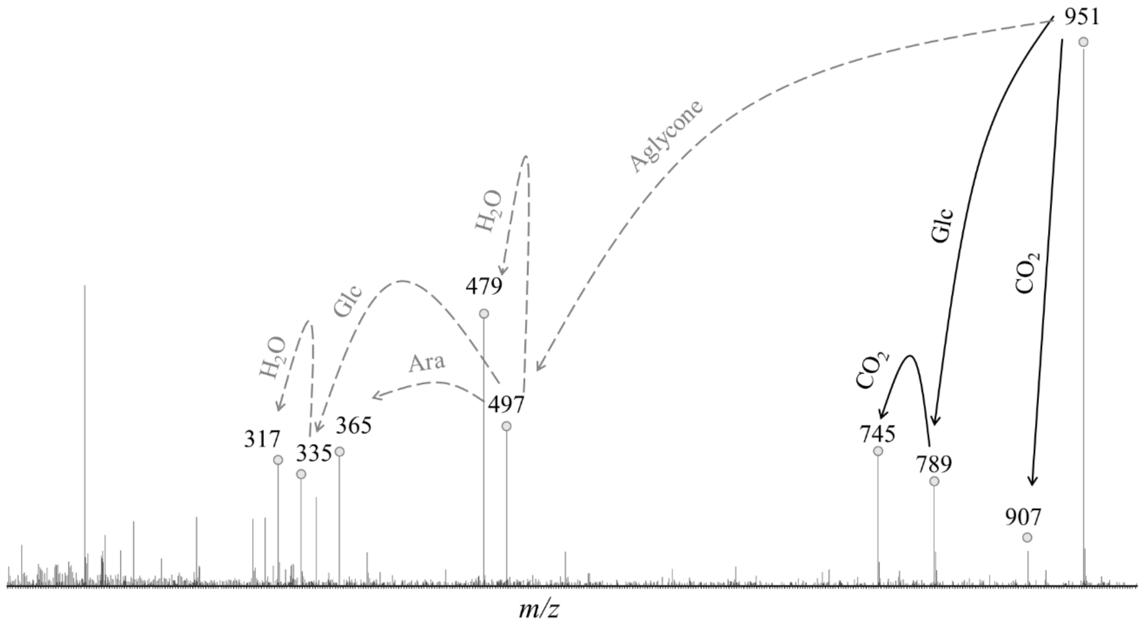

Figure S1u: LC-MSMS analysis of *Chenopodium quinoa* husk saponin extract: CID spectrum recorded for the *m/z* 951 precursor ions at 10.2 min retention time. The corresponding ions are assigned as $[M+Na]^+$ ions from Saponin 61[h].

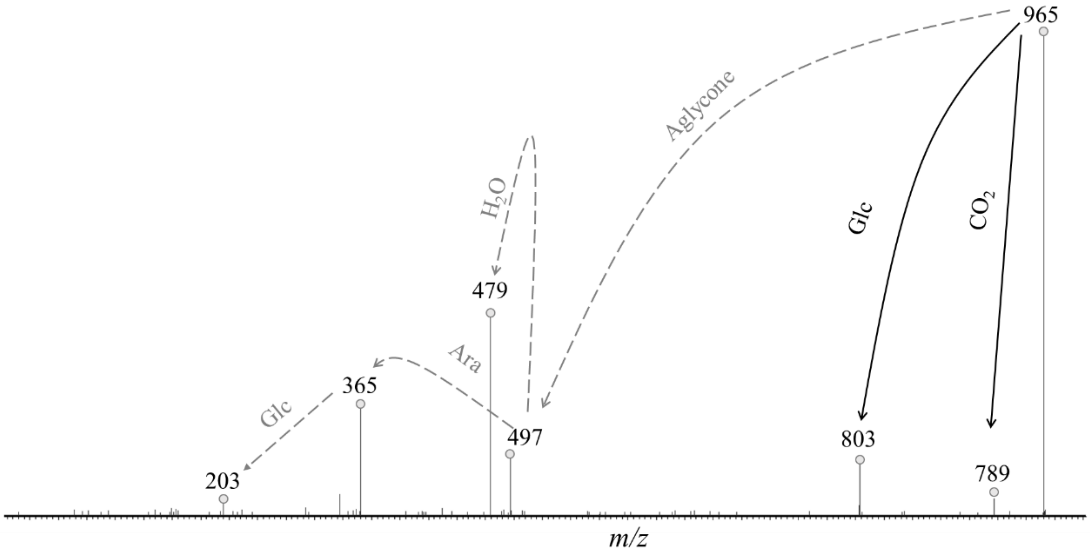

Figure S1v: LC-MSMS analysis of ***Chenopodium quinoa*** husk saponin extract: CID spectrum recorded for the *m/z* 965 precursor ions at 7 min retention time. The corresponding ions are assigned as $[M+Na]^+$ ions from Saponin ??[h].

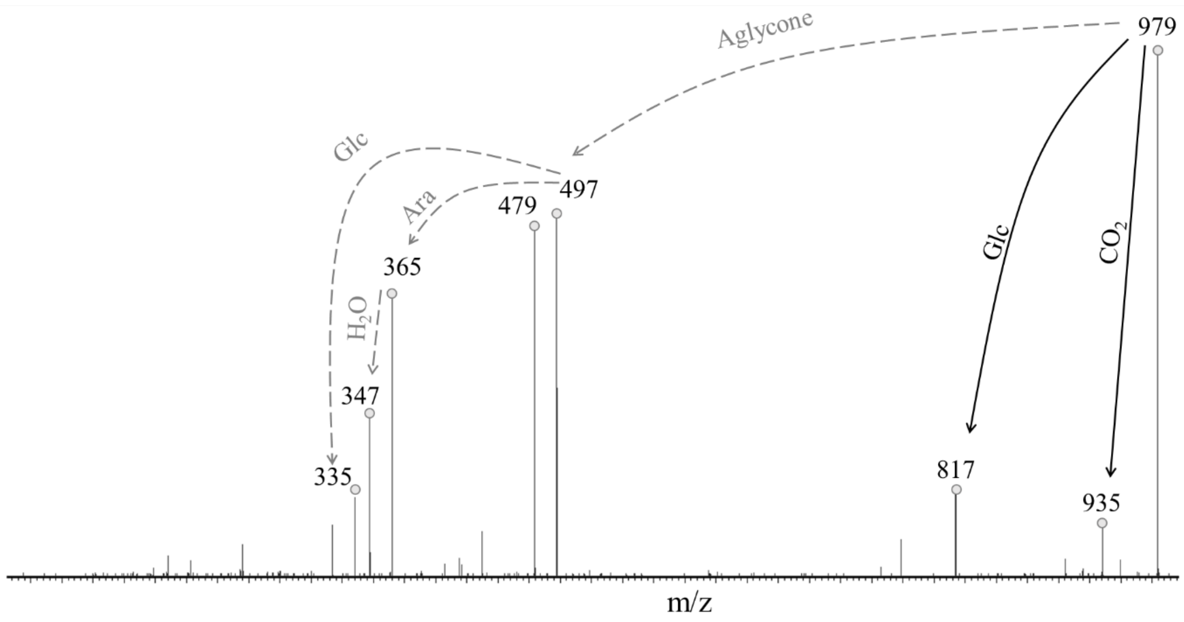

Figure S1w: LC-MSMS analysis of *Chenopodium quinoa* husk saponin extract: CID spectrum recorded for the *m/z* 979 precursor ions at 9.4 min retention time. The corresponding ions are assigned as [M+Na]+ ions from Saponin Gh.

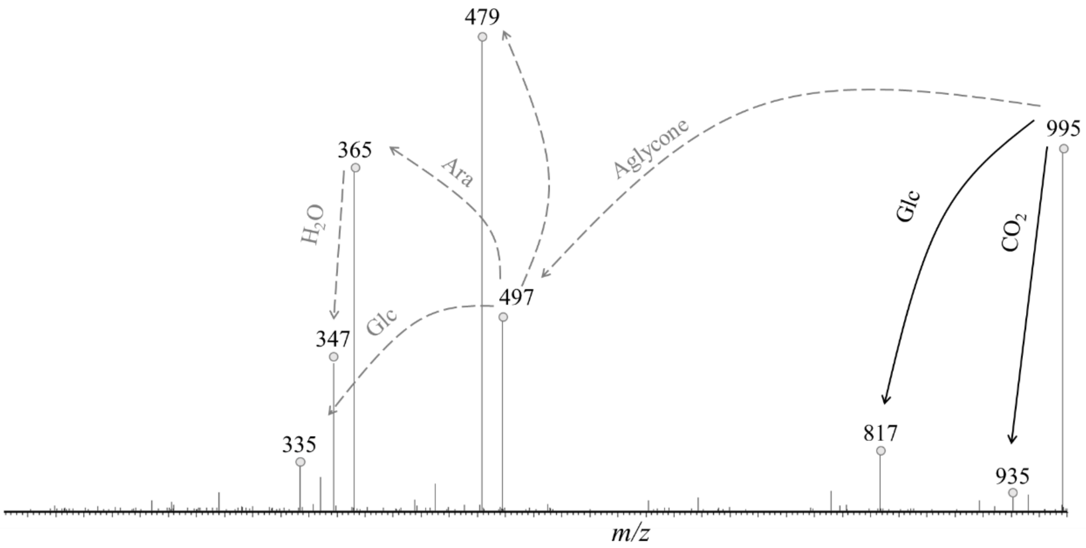

Figure S1x: LC-MSMS analysis of *Chenopodium quinoa* husk saponin extract: CID spectrum recorded for the *m/z* 995 precursor ions at 7.6 min retention time. The corresponding ions are assigned as [M+Na]+ ions from Saponin Oh.

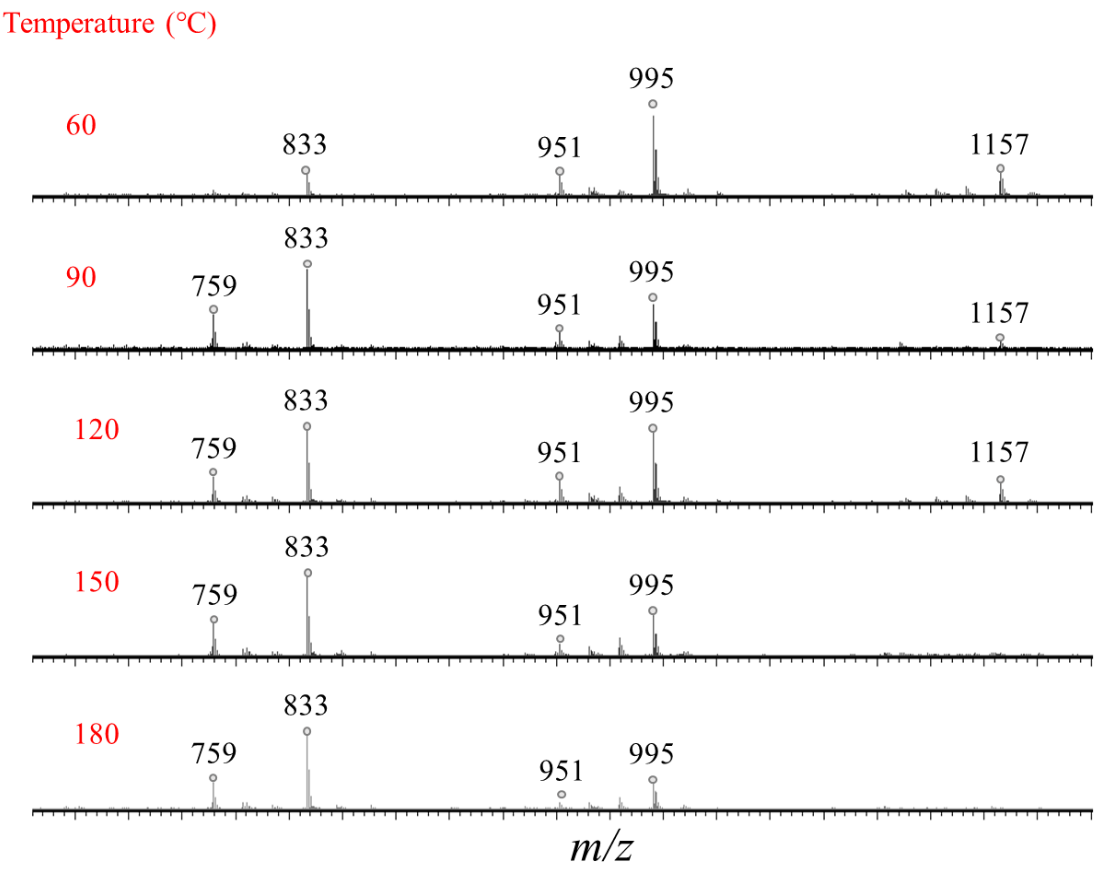

Figure S2: Microwave-assisted hydrolysis of the quinoa bidesmosidic saponins (5 min at pH 11): influence of the temperature (60,90, 120, 150, 180°C) on hydrolysis reactions from quinoa husk saponin determined by MALDI-MS.

Table S1: Hemolytic activity assay of the hydrolyzed and natural extract saponin. Average and standard deviation of the free heme absorbance (540 nm) with regards to the increasing saponin concentration.

Concentration ($\mu g.ml^{-1}$)	Absorbance (450 nm) of natural extract saponin		Absorbance (450 nm) of Hydrolyzed saponin	
	Average	Standard deviation	Average	Standard deviation
0.5	0.0163	0.0021	0.0106	0.0008
1	0.0123	0.0029	0.0012	0.0042
2	0.0143	0.0048	0.0012	0.0015
3	0.0134	0.0041	0.0008	0.0065
4	0.0106	0.0030	0.0005	0.0018
5	0.0116	0.0036	0.0014	0.0012
10	0.0109	0.0027	0.0016	0.0046
20	0.0097	0.0136	0.0005	0.0039
30	0.0083	0.0042	0.0002	0.0007
40	0.0075	0.0012	0.0009	0.0069
50	0.0067	0.0003	0.0007	0.0005
100	0.0050	0.0050	0.0314	0.0004
200	0.0066	0.0071	0.3210	0.0130
300	0.0036	0.0008	0.7210	0.0812
500	0.0034	0.0018	/	/
